# Exploring the Effects of the Spatial Distribution of Catalytic Sites on Sulfur Nucleation Behaviors and Electrochemical Performances of Lithium–Sulfur Batteries

**DOI:** 10.1002/advs.202513026

**Published:** 2025-09-26

**Authors:** Shin‐Yeong Kim, Hyeonwoo Cho, Seong‐Jun Kim, Minchul Ahn, Yunseo Jeoun, Kookhan Kim, Sung‐Pyo Cho, So Hee Kim, Gi Young Son, Seung‐Ho Yu, Byung Hee Hong, Yung‐Eun Sung

**Affiliations:** ^1^ Department of Chemical and Biological Engineering Seoul National University Seoul 08826 Republic of Korea; ^2^ Center for Nanoparticle Research Institute for Basic Science (IBS) Seoul 08826 Republic of Korea; ^3^ Department of Chemistry Seoul National University Seoul 08826 Republic of Korea; ^4^ Graphene Research Center Advanced Institute of Convergence Technology Suwon 16229 Republic of Korea; ^5^ Department of Chemical and Biological Engineering Korea University Seoul 02841 Republic of Korea; ^6^ Chemical Materials R&D Department Korea Automotive Technology Institute Cheonan Chungnam 31214 Republic of Korea; ^7^ National Center for Inter‐University Research Facilities Seoul National University Seoul 08826 Republic of Korea; ^8^ Advanced Analysis Center Korea Institute of Science and Technology (KIST) Seoul 02792 Republic of Korea; ^9^ Department of Battery‐Smart Factory Korea University Seoul 02841 Republic of Korea

**Keywords:** graphene quantum dots, high donor electrolytes, lithium‐sulfur batteries, nucleation behaviors, spatial distributions

## Abstract

Achieving 3D Li_2_S morphologies and accelerating polysulfide conversion reactions are critical for realizing high‐energy lithium–sulfur (Li–S) batteries. Although electrolytes containing high Gutmann donor number components facilitate transition from film‐like to 3D Li_2_S morphologies, challenges arising from polysulfide diffusion and incomplete polysulfide conversion remain unresolved. Catalyst design strategies optimized for high donor electrolyte systems are essential in overcoming these limitations, yet they have garnered limited attention. Here, the influence of catalytic site distribution is systematically investigated as a key variable in catalyst design principles for high donor systems, aiming to achieve high‐capacity and stable Li–S battery operation. Two types of carbon model systems are designed and employed in Li–S cells. One type features widespread distribution of catalytic sites, whereas the other incorporates localized catalytic sites. Experimental results demonstrate that spatial confinement of catalytic sites facilitates effective polysulfide redox reactions and supports the formation of 3D Li_2_S morphologies, even more pronounced in high donor electrolytes, thereby achieving near‐theoretical capacity of sulfur (1630 mA h g^−1^) and ensuring stable cycling. These findings highlight that spatial control of catalytic sites is a key parameter for optimizing next‐generation Li–S battery performances, offering novel design principles for advanced battery systems.

## Graphical abstract

The effects of catalytic site distributions on Li_2_S nucleation behaviors and electrochemical performances in lithium–sulfur batteries are systematically explored. This study demonstrates how tailoring the spatial arrangement of catalytic sites can control sulfur deposition behavior, leading to enhanced battery performances. The findings offer new catalyst design principles for Li–S battery systems utilizing advanced electrolytes.

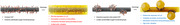

## 1 Introduction

As the demands for rechargeable batteries with high energy densities continue to grow, extensive research efforts are being directed toward the development of next‐generation battery systems beyond lithium‐ion batteries. Owing to the high theoretical gravimetric capacities of elemental sulfur and metallic lithium (1675 and 3860 mA h g^−1^, respectively), lithium–sulfur (Li–S) batteries have emerged as promising candidates for advanced energy storage systems, with the potential to achieve a practical gravimetric energy density of up to 500 W h kg^−1^.^[^ ^1^ ^]^ Furthermore, due to its natural abundance and environmental friendliness, elemental sulfur is regarded as an attractive cathode active material. However, the practical viability of Li–S batteries is hindered by several critical challenges, including polysulfide shuttling, insulating nature of sulfur species (i.e., S_8_ and Li_2_S), and surface passivation of lithium (Li) metal anode.^[^ ^2^, ^3^ ^]^ Because of these unresolved fundamental limitations, the path toward the commercialization of Li–S batteries demands continued technological breakthroughs. In particular, the formation of inherently insulating Li_2_S during the discharge process significantly increases cell resistance, which adversely affects sulfur utilization and compromises cycle life.^[^ ^2^, ^3^ ^]^ The film‐like growth of Li_2_S is known to exacerbate the loss of conductivity during battery cycling by passivating the surface of conductive materials.

To mitigate insulation induced by film‐like Li_2_S, various studies have aimed to promote 3D growth of Li_2_S, thereby suppressing Li_2_S film formation and preventing the passivation of conductive materials.^[^ ^4^, ^5^ ^]^ The growth of 3D Li_2_S particles is facilitated by solvent‐mediated processes,^[^ ^3^, ^4^, ^6^, ^7^, ^8^, ^9^ ^]^ and particle‐shaped catalysts have also been reported to preferentially induce the formation of Li_2_S with 3D morphologies.^[^ ^10^, ^11^, ^12^, ^13^ ^]^ In particular, electrolytes containing high Gutmann donor number (DN) components enable the solvent‐mediated processes, leading to high sulfur utilization due to increased polysulfide solubility and a new reaction pathway involving stabilized sulfur radical intermediates (i.e., S_3_ **^•−^**), which act as redox mediators.^[^ ^4^, ^14^ ^]^ As Li–S batteries utilizing high donor electrolytes have demonstrated promising performances in recent studies, their potential for practical application has garnered growing attention. However, even with the use of high donor electrolytes in place of conventional ether‐based electrolytes, the migration of polysulfides to the Li metal anode surface and shuttling of polysulfides persist.^[^ ^4^ ^]^ To address these challenges, the integration of polysulfide‐adsorbable catalysts in high donor electrolyte systems appears to be a promising strategy for future practical applications of Li–S batteries. However, this strategy remains largely unexplored, and the key variables in catalyst design for realizing high‐performance cells are still not well defined.

To evaluate the effectiveness of catalyst integration strategy, we constructed novel model systems using functionalized carbon–based materials, which are widely employed in fundamental catalytic studies of Li–S batteries.^[^ ^15^, ^16^, ^17^, ^18^ ^]^ In particular, catalyst design strategies in the Li–S battery research have primarily focused on enhancing catalytic activity by considering only single‐molecule reactions, analogous to approaches used in oxygen reduction reaction electrocatalyst studies. Instead, we emphasized the role of Li_2_S nucleation processes during the liquid‐to‐solid conversion reactions in Li–S batteries. We hypothesized that, similar to particle‐shaped catalysts, spatial control of catalytic sites could critically regulate nucleation behaviors through modulation of polysulfide adsorption site geometry. To investigate how the spatial distributions of functional groups affect the Li_2_S nucleation mechanisms, we employed graphene quantum dots (GQDs). GQDs are 0D carbon nanomaterials derived from graphene, and have been widely applied in energy storage devices owing to their large surface area, which facilitates both physicochemical adsorption and rapid electron transfer.^[^ ^17^, ^19^, ^20^, ^21^, ^22^ ^]^ Noting that GQDs possess a 0D structure, we speculated that they could be utilized to construct model materials incorporating localized functional groups. Herein, oxidized multiwalled carbon nanotubes (O*x*CNT), synthesized by a mild chemical oxidation method, and multiwalled carbon nanotubes loaded with oxygen‐functional‐group‐rich GQDs (CNT/GQD) substrates were adopted to modulate spatial distributions of oxygen‐containing functional groups on multiwalled carbon nanotube (MWCNT) surfaces. By introducing model systems with comparable oxygen contents but distinct spatial distributions of oxygen functional groups, we elucidated how the distribution of polysulfide adsorption site influences Li_2_S nucleation behaviors. Experimental results revealed that, due to the uniformly distributed oxygen functional groups on O*x*CNT, Li_2_S exhibited film‐like morphologies, which led to continuous capacity fading in conventional ether‐based electrolyte systems. In contrast, the locally concentrated oxygen functional groups introduced by GQDs promoted 3D Li_2_S deposition, which is attributed to the restricted spatial distribution of adsorption sites. The designed catalyst exhibited stable cycling performances and enhanced reaction kinetics. In addition, when using an electrolyte based on 1,3‐dimethyl‐2‐imidazolidinone (DMI), a high DN solvent known for its excellent electrochemical performances,^[^ ^4^ ^]^ Li_2_S nucleation behaviors similar to those in ether‐based electrolyte systems were observed. In DMI‐based electrolytes, catalysts with spatially confined catalytic sites demonstrated near‐theoretical capacities (1630 mA h g^−1^), highlighting that catalyst‐integration strategies remain critical even under advanced electrolyte environments. Moreover, our model system approach clarifies the correlation between 3D deposition behavior and the resulting performance enhancements—a relationship that has remained unclear due to variations in surface functionalization of catalytic materials and changes in solvation structure induced by electrolyte engineering. This study provides new insights into how the spatial distributions of adsorption sites in sulfur reduction electrocatalysts govern the morphologies of deposited Li_2_S, and how catalyst design strategies can be formulated to enhance electrochemical performances in both ether‐based electrolytes and high donor electrolytes.

## 2 Results and Discussion

### 2.1 Synthesis and Characterization of GQDs

**Figure 1:**
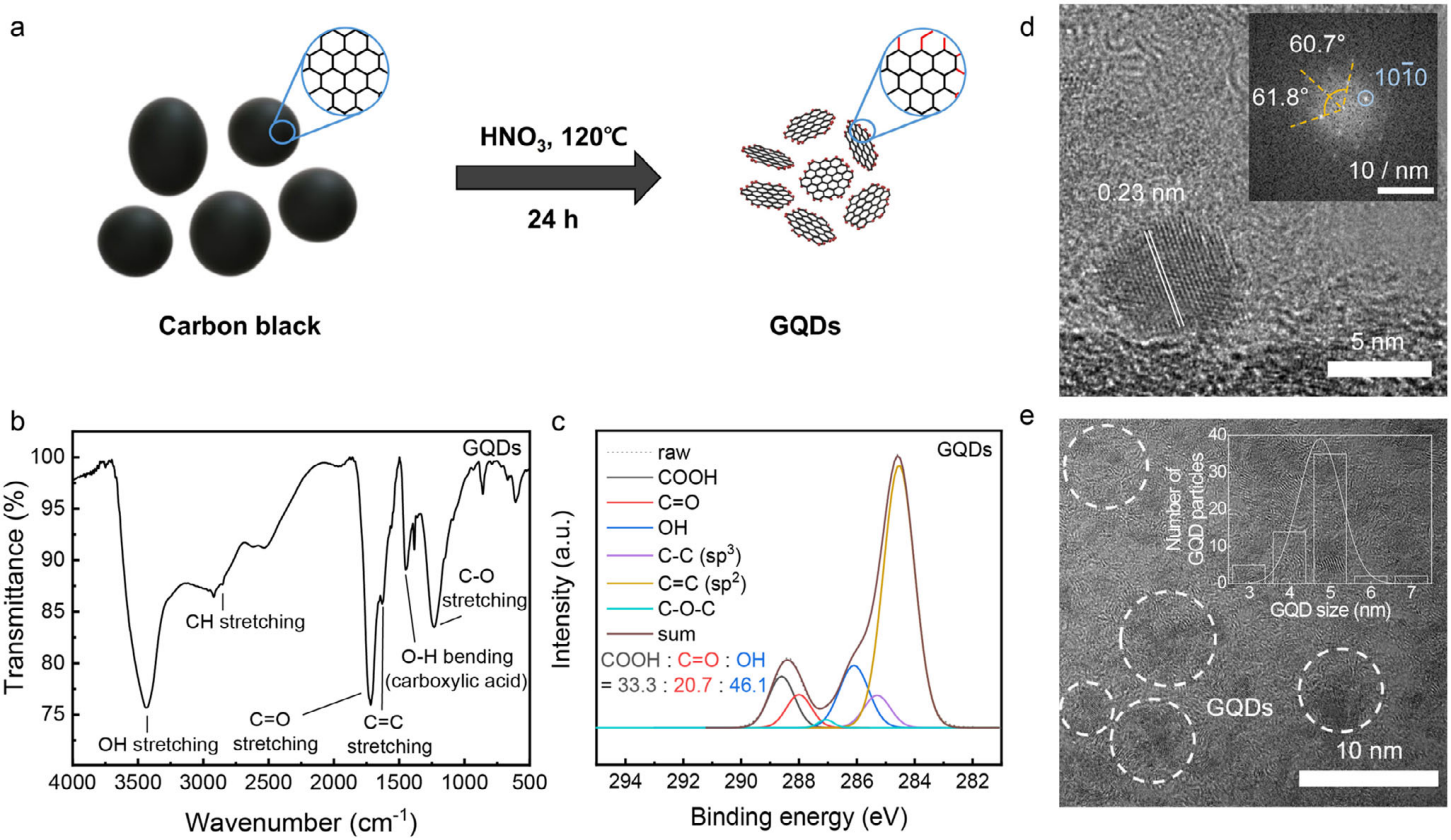
Characterization of GQDs. a) Schematic diagram of synthetic procedures of GQDs. b) FT‐IR spectrum of GQDs. c) High‐resolution C 1s XPS spectrum of GQDs. d) High magnification Cs‐TEM image of GQDs, with an inset showing the corresponding FFT. e) Low magnification Cs‐TEM image of GQDs, with an inset showing the corresponding particle size distribution.

Oxygen‐rich GQDs were synthesized via a top‐down oxidative‐cutting method, in which carbon black was cleaved in concentrated nitric acid, followed by thorough purification to remove residual reagents (**Figure** **1**a).^[^ ^23^, ^24^, ^25^ ^]^ The resulting GQDs possess abundant functional groups at their edges, while retaining a graphene lattice structure in the core region. The FT‐IR spectrum of the GQDs (Figure 1b) shows a characteristic aromatic C═C stretching vibration at 1629.5 cm^−1^, along with absorption bands attributable to oxygen‐containing functional groups: O─H stretching (3440 cm^−1^), C═O stretching (1720 cm^−1^), C─O stretching (1230 cm^−1^), and carboxylic O─H bending (1440 cm^−1^). Elemental analysis confirmed that the synthesized GQDs possess a high oxygen content of 49.5 wt% (Table S1, Supporting Information). High‐resolution XPS of the C 1s region (Figure 1c) further reveals the significant presence of COOH (288.5 eV), C═O (288.0 eV), and C─O─H (286.1 eV) species,^[^ ^26^, ^27^ ^]^ indicating the high density of oxygen‐rich moieties in GQDs. These rich surface functionalities considerably enhance sulfiphilicity, as evidenced by superior Li_2_S_6_ adsorption compared to conventional carbon materials (Figure S1, Supporting Information).

Atomic‐resolution imaging by spherical aberration–corrected transmission electron microscopy (Cs‐TEM; Figure 1d,e; Figure S2a, Supporting Information) reveals a lattice fringe spacing of ≈0.23 nm, consistent with (10$\bar{1}$0) plane of graphene,^[^ ^28^ ^]^ and confirmed by fast Fourier transform (FFT) patterns showing a hexagonal lattice composed of sp^2^‐hybridized carbon atoms with ≈60° inter‐spot angles.^[^ ^29^, ^30^ ^]^ The Raman spectrum (Figure S2b, Supporting Information) exhibits both D (1350 cm^−1^) and G (1580 cm^−1^) bands with an intensity ratio (I_D_/I_G_) of 0.94, corroborating the presence of graphene frameworks in GQDs. Low magnification Cs‐TEM image reveals an average lateral dimension of ≈5 nm, while the dynamic light scattering (DLS) result demonstrates a monomodal hydrodynamic size distribution, confirming narrow particle size distribution of the synthesized GQDs (Figure 1e; Figure S2a,c, Supporting Information).

### 2.2 Synthesis and Characterization of CNT/GQDs and O*x*CNTs

**Figure 2:**
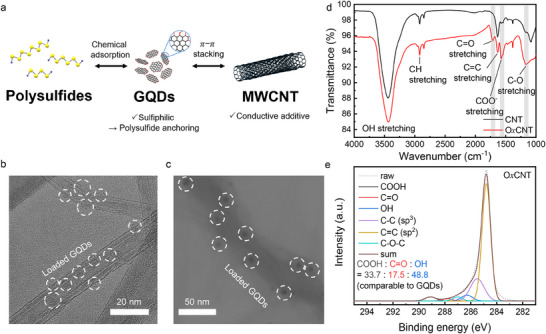
Characterization of CNT/GQDs and OxCNTs. a) Schematic representation of the material design for CNT/GQD composites. b) Cs‐TEM and c) BF‐TEM images showing GQDs loaded on MWCNTs. d) FT‐IR spectra of CNT and OxCNT. e) High‐resolution C 1s XPS spectrum of OxCNT.

CNT/GQDs were prepared by noncovalently loading GQDs onto MWCNTs through π–π interactions between their graphene lattice structures, while the abundant oxygen‐containing functional groups on the GQDs further facilitate chemical adsorption and anchoring of polysulfides (**Figure** **2**a–c; Figure S3, Supporting Information). We utilized the intrinsic fluorescence of GQDs to quantify their maximum loading amounts on MWCNTs (Figure S4a, Supporting Information).^[^ ^31^ ^]^ Upon centrifugation after the co‐dispersion of MWCNTs and GQDs in deionized water, unbound GQDs remain in the supernatant, exhibiting a fluorescence emission peak at 560 nm under 360 nm excitation (Figure S4b, Supporting Information). To quantify the amount of unbound GQDs in the supernatant, a calibration curve (R^2^ = 0.9987) was established by plotting fluorescence intensities at 560 nm as a function of GQD concentrations (Figure S4c, Supporting Information). By adding GQDs into MWCNT dispersions, we found that the loading saturates at a MWCNT:GQD weight ratio of 10:1 (Figure S4d, Supporting Information), corresponding to a maximum GQD content of 10 wt% relative to MWCNTs. The loading amount of GQDs on MWCNTs does not exceed 10 wt%, which is attributed to a balance between hydrogen bonding of GQDs with water and π–π interactions at the GQD–MWCNT interface. CNT/GQD composites were prepared at 10:1 weight ratio, and the successful attachment of GQDs on MWCNTs was confirmed by Cs‐TEM and bright‐field transmission electron microscopy (BF‐TEM) imaging (Figure 2b,c; Figure S3d, Supporting Information).

Based on elemental analysis results of GQDs and MWCNTs, we estimated the oxygen content in CNT/GQD composites and subsequently prepared O*x*CNTs possessing a comparable oxygen loading of ≈6 wt%, thereby minimizing activity differences arising from inconsistencies in oxygen contents (Table S1, Supporting Information). A mild chemical oxidation protocol^[^ ^32^, ^33^ ^]^ was chosen to introduce uniformly distributed oxygen functionalities, while preserving much of the intrinsic graphene lattice.

The FT‐IR spectra (Figure 2d) confirm the successful oxidation of O*x*CNTs: pristine MWCNTs exhibit C═C stretching at 1630 cm^−1^ (with the broad O─H band at ≈3200–3600 cm^−1^ primarily arising from adsorbed moisture),^[^ ^34^, ^35^ ^]^ whereas O*x*CNTs display additional bands at 1710 cm^−1^ (C═O), 1573 cm^−1^ (COO^−^), and 1165 cm^−1^ (C─O). High‐resolution C 1s XPS analysis in Figure 2e further resolves contributions from COOH (289.1 eV), C═O (287.8 eV), C─O─C (287.0 eV), and C─OH (286.3 eV), with O*x*CNTs comprising 33.3% COOH, 20.7% C═O, and 46.1% OH, closely matching the distribution observed in GQDs (Figure S5, Table S2, and Note S1, Supporting Information). This matching of oxygen functional group ratios further minimizes electrocatalytic activity differences arising from the individual activity of each functional group, thereby confirming that CNT/GQD and O*x*CNT serve as rational model systems in which only the spatial distributions of oxygen‐containing active sites are systematically controlled. The enriched surface chemistries of O*x*CNTs yields improved Li_2_S_6_ adsorption (Figure S1, Supporting Information), attesting to enhanced sulfiphilicity of oxidized carbon materials. The transmission electron microscopy (TEM) image of O*x*CNTs verifies that the tubular morphologies remain intact after oxidation processes (Figure S6, Supporting Information).

N_2_ adsorption–desorption isotherms (Figure S7a–c, Supporting Information) reveal Brunauer–Emmett–Teller (BET) surface areas of 185.3, 242.0, and 199.2 m^2^ g^−1^ for MWCNTs, O*x*CNTs, and CNT/GQDs, respectively. The modest increase in surface area for O*x*CNTs and CNT/GQDs are attributed to oxidative cutting and the presence of nanoscale GQDs, yet the values remain within a narrow range. Barrett–Joyner–Halenda (BJH) pore‐size distributions (Figure S7d–f, Supporting Information) show abundant macropores (>50 nm) originating from intertube spacing, which facilitate rapid polysulfide diffusion.^[^ ^36^ ^]^ These void‐rich structures suggest that the CNT‐based substrates enable efficient mass transport and serve as model systems where electrochemical performances are predominantly governed by the surface properties of the catalysts.

Together, these findings confirm that the resulting CNT/GQD and O*x*CNT materials serve as robust model systems for studying catalysts with distinct spatial distributions of active sites. In CNT/GQDs, oxygen functional groups are densely and locally concentrated within nanosized (≈5 nm) graphene quantum dots, whereas in O*x*CNTs, the oxygen functional groups are uniformly distributed along the CNT tubes, leading to a clear difference in spatial distribution of oxygen functional groups.

### 2.3 Nucleation Behaviors in Conventional DOL/DME‐Based Electrolytes

**Figure 3:**
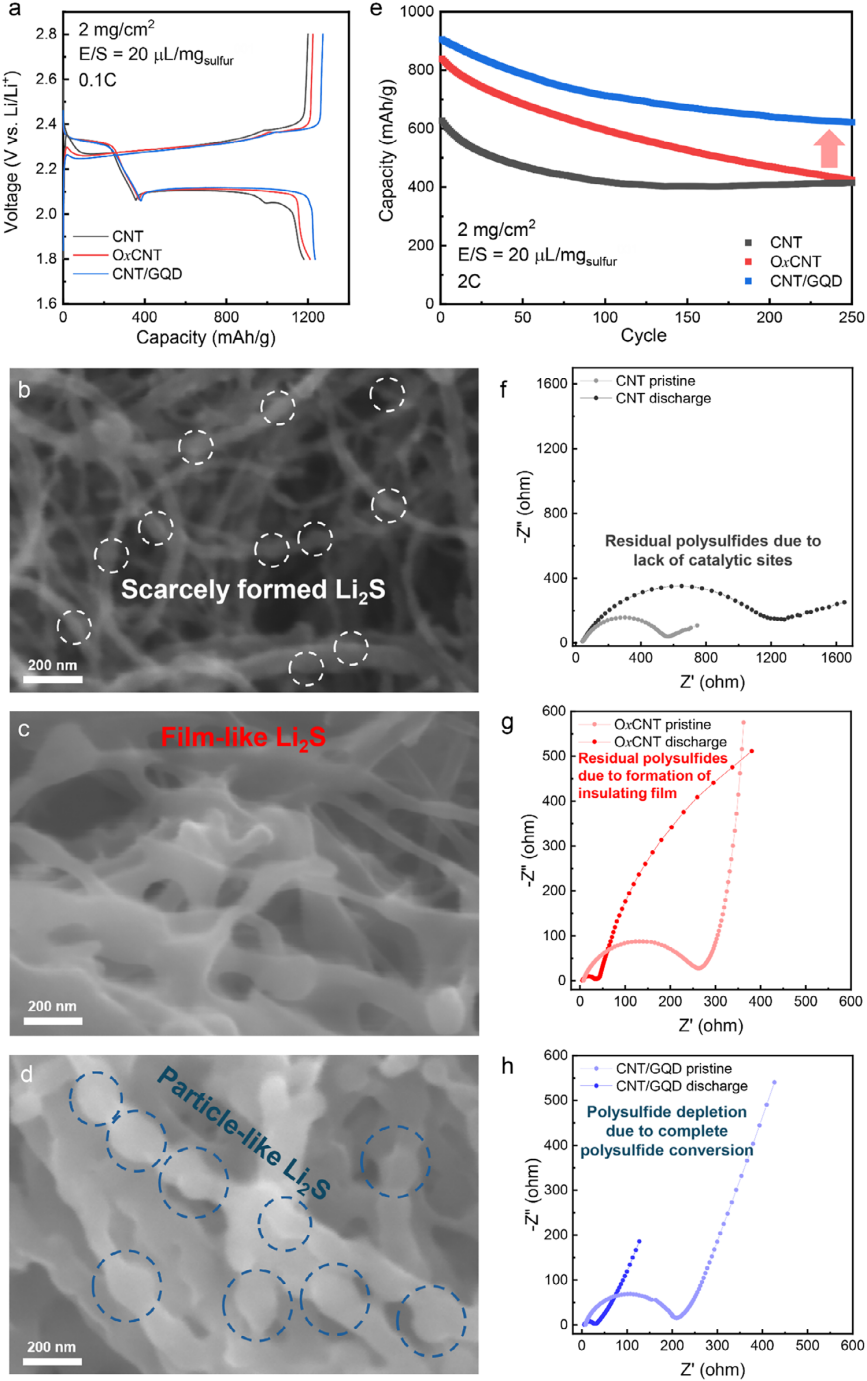
Investigation of nucleation behaviors and electrochemical performances in Li–S cells using DOL/DME‐based electrolytes. a) First galvanostatic charge‐discharge profiles of Li–S cells with DOL/DME‐based electrolytes at 0.1C. Ex situ SEM images of Li_2_S deposits on b) CNT, c) OxCNT, d) CNT/GQD substrates. e) Cycling performances of cells with different carbon substrates at 2C. In situ EIS of f) CNT, g) OxCNT, h) CNT/GQD.

To investigate the effects of spatial distributions of catalytic sites on Li_2_S nucleation and electrochemical behaviors of Li–S cells, sulfur cathodes were prepared using each carbon substrate, and their electrical conductivities were determined by four‐point probe measurements (Table S2, Supporting Information). Although the introduction of oxidized carbon materials (GQDs and O*x*CNTs) marginally reduced electrical conductivities, the differences were negligible; thus, the electrochemical performance differences can be primarily ascribed to surface chemistries rather than bulk electronic conductivities. **Figure** **3**a shows the voltage profiles at 0.1C when commercial DOL/DME‐based electrolytes were used. The pristine MWCNT cathode delivered a discharge capacity of ≈1050 mA h g^−1^, whereas O*x*CNT and CNT/GQD achieved high capacities of ≈1201 and ≈1253 mA h g^−1^, respectively. These results reflect enhanced sulfur conversion kinetics enabled by surface oxygen groups, in agreement with cyclic voltammetry (CV) results (Figure S8, Supporting Information). Notably, CNT/GQD exhibited lower overpotential for Li_2_S conversion reactions in CV tests and lower charging onset potential for Li_2_S decomposition than O*x*CNT (Figures S8 and S9, Supporting Information), despite the formation of a larger quantity of insulating Li_2_S in CNT/GQD cathodes. These results suggest that Li_2_S nucleation on CNT/GQD is kinetically more favorable, and that such distinct nucleation behavior contributes to mitigating conductivity losses associated with thick Li_2_S films.^[^ ^9^, ^37^, ^38^, ^39^, ^40^ ^]^

Ex situ SEM characterizations of discharged electrodes (Figure 3b–d) were performed under inert transfer to preserve Li_2_S morphologies. On pristine MWCNT substrates, nucleated Li_2_S was sparse and exhibited film‐like morphologies, due to the limited available catalytic sites (Figure 3b). This observation aligns with prior reports, where limited nucleation is commonly observed on catalytically inert surface.^[^ ^4^ ^]^ In contrast, distinct nucleation behaviors were observed on O*x*CNT and CNT/GQD substrates (Figure 3c,d). For O*x*CNT, thick and continuous Li_2_S films were formed, likely increasing interfacial resistance due to the insulating nature of Li_2_S. Such film‐like deposition of Li_2_S is typical for oxidized carbon surfaces and is consistent with previously reported findings.^[^ ^41^ ^]^ On the other hand, the CNT/GQD substrates exhibited markedly different morphologies. While regions of bare MWCNTs were partially coated by Li_2_S films, GQD‐decorated areas showed the formation of discrete, 3D Li_2_S particles (≈100 nm in diameter). Given the higher polysulfide affinity of GQD regions relative to bare MWCNT regions, these results suggest that GQDs promote spatially confined and particle‐like Li_2_S nucleation, which can mitigate electrode passivation and maintain ionic/electronic conductivity. This behavior is attributed to the concentrated presence of oxygen‐containing functional groups on GQDs, as opposed to the uniformly dispersed functional groups on O*x*CNT. In O*x*CNT, the continuous Li_2_S films act as passivation layers, resulting in poor reversibility and the accumulation of residual Li_2_S over cycling.

To correlate these morphological observations with electrochemical performances, galvanostatic cycling was conducted at a rate of 2C (Figure 3e). Both O*x*CNT and CNT/GQD exhibited higher initial capacities (≈830 and ≈900 mA h g^−1^, respectively) compared to bare CNT cathodes (≈620 mA h g^−1^), which is attributed to surface oxygen functionalization. Nonetheless, capacity retention varied significantly: O*x*CNT showed the highest degradation rate (0.20% per cycle), followed by MWCNT (0.14% per cycle), while CNT/GQD exhibited the most stable performance (0.12% per cycle), despite delivering the highest initial capacity in CNT/GQD. The observed differences in degradation tendencies reflect distinct degradation mechanisms under the high‐rate condition of 2C. For MWCNT, early‐cycle degradation is primarily ascribed to polysulfide dissolution and diffusion toward Li metal anode. While O*x*CNTs effectively immobilize polysulfides through adsorption, gradual capacity fading occurs during cycling as insulating Li_2_S films accumulate. Notably, MWCNT showed rapid degradation within the first 50 cycles, after which the capacity plateaued, whereas O*x*CNT exhibited a more gradual, yet continuous, decay up to 250 cycles. In the case of CNT/GQD, both degradation behaviors were effectively resolved, underscoring that the spatially distributed oxygen functional groups on CNT/GQD substrates contribute to improved reversibility and long‐term stability by enabling polysulfide immobilization and promoting a particle‐like Li_2_S nucleation pathway. Moreover, our results based on the use of model systems, in which catalytic activity differences were controlled at the atomic level, provide a rational basis for elucidating the correlation between Li_2_S deposition behaviors and electrochemical performances.

Electrochemical impedance spectroscopy (EIS) was conducted to further probe the correlation between nucleation mode and charge‐transfer kinetics (Note S2, Supporting Information). For MWCNT (Figure 3f), the charge transfer resistance increased during the polysulfide conversion. However, a persistent semicircle in the Nyquist plot was still observed, indicating that residual unconverted polysulfides and limited catalytic activity.^[^ ^42^ ^]^ This result suggests that MWCNT exhibits sluggish conversion reaction kinetics due to a lack of catalytic sites. Similarly, in O*x*CNT (Figure 3g), a larger semicircle appeared, reflecting increased resistance. By contrast, CNT/GQD (Figure 3h) exhibited no discernible semicircle even at low frequencies down to 10 mV, implying that the charge transfer resistance exceeded the measurement range. This result indicates near‐complete conversion of polysulfides to Li_2_S, with minimal remaining polysulfide intermediates.^[^ ^3^, ^42^ ^]^ However, in the case of O*x*CNT, the presence of a partial semicircle suggests the accumulation of residual polysulfides, implying incomplete conversion to Li_2_S and conductivity loss caused by Li_2_S film–induced passivation. These findings collectively demonstrate that the particle‐like Li_2_S deposition enabled by CNT/GQD not only enhances polysulfide conversion kinetics but also prevents electrode passivation, leading to superior electrochemical performances.

### 2.4 Nucleation Behaviors in High Donor Electrolytes

Li_2_S nucleation behaviors on different carbon substrates were further investigated using a DMI‐based electrolyte, known for its high DN and strong compatibility with Li metal anode.^[^ ^4^, ^43^ ^]^ High donor electrolytes have demonstrated notable electrochemical performances in Li–S batteries. A distinguishing feature of these systems is their ability to induce the direct formation of particle‐like Li_2_S on carbon surfaces without the need for additional surface functionalization. Owing to this unique Li_2_S nucleation mechanism, Li–S cells employing high donor electrolytes can deliver high capacities and are currently under active investigation for commercial viability, particularly under lean electrolyte conditions.^[^ ^44^, ^45^, ^46^, ^47^ ^]^ Nevertheless, similar to ether‐based electrolytes, the dissolution and diffusion of polysulfide species remain problematic, necessitating strategies that immobilize polysulfides and enhance their conversion kinetics via functional group modification of carbon substrates. However, there have been no reported studies investigating the effect of functionalized carbon substrates in high donor electrolytes. To bridge this gap, we carried out a comparative study on the nucleation behaviors and electrochemical performances of carbon model systems using DMI‐based high donor electrolytes.

**Figure 4:**
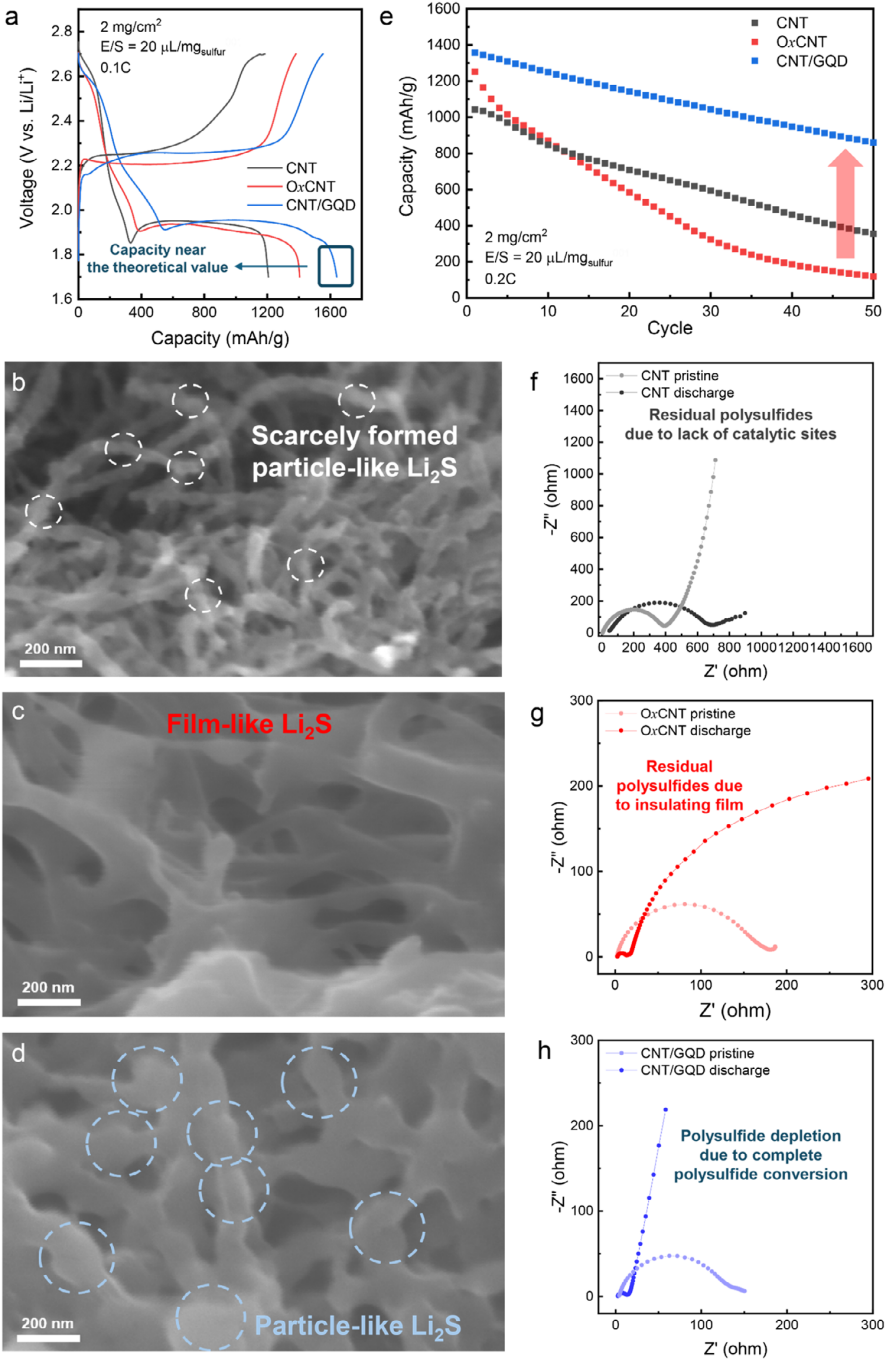
Investigation of nucleation behaviors and electrochemical performances in Li–S cells using high donor electrolytes. a) First galvanostatic charge‐discharge profiles of Li–S cells with DMI‐based electrolytes at 0.1C. Ex situ SEM images of Li_2_S deposits on b) CNT, c) OxCNT, d) CNT/GQD substrates. e) Cycling performances of cells with different carbon substrates at 0.2C. In situ EIS of f) CNT, g) OxCNT, h) CNT/GQD.

**Figure** **4**a shows the voltage profiles of the different carbon substrates. CNT/GQD (≈1630 mA h g^−1^) exhibited a higher capacity than O*x*CNT (≈1405 mA h g^−1^), approaching the theoretical capacity of sulfur (1675 mA h g^−1^). Ex situ SEM was employed to visualize Li_2_S morphologies. As previously reported, particle‐like Li_2_S formation was observed on pristine MWCNTs,^[^ ^4^ ^]^ but Li_2_S formation was limited due to the scarcity of catalytic sites (Figure 4b). Interestingly, on O*x*CNT substrates, Li_2_S was found to grow in a film‐like manner, analogous to the behavior observed in ether‐based electrolytes (Figure 4c). This observation suggests that the charge‐transfer‐controlled driving force for Li_2_S film formation, induced by polysulfide immobilization via oxygen functional groups, could surpass the mass‐transfer‐controlled driving force for 3D Li_2_S growth, which is driven by solvent‐mediated processes of high donor electrolytes. This behavior is consistent with the suggested nucleation mechanisms of the recent study.^[^ ^48^ ^]^ In contrast, the CNT/GQD substrates retained particle‐like Li_2_S morphologies (Figure 4d), effectively mitigating conductivity loss–induced kinetic limitations and enabling high capacity. To compare the amount of Li_2_S formed in ether‐based and high donor electrolytes, ex situ X‐ray diffraction (XRD) analysis was conducted at the discharged state (Figures S10 and S11, Supporting Information). For both O*x*CNT and CNT/GQD electrodes, the XRD patterns exhibited more pronounced Li_2_S diffraction peaks in the high donor electrolyte, indicating enhanced Li_2_S formation.^[^ ^49^, ^50^, ^51^ ^]^ These results suggest that, despite the larger amount of insulating Li_2_S, its formation in a 3D morphology prevented conductivity losses, enabling efficient deposition. To obtain additional kinetic and mechanistic insights into nucleation, symmetric CV and chronoamperometry tests were performed, which further confirmed the favorable nucleation kinetics of CNT/GQD (Figures S12–S14 and Notes S3 and S4, Supporting Information).

Cycle stabilities of carbon substrates in high donor electrolytes are presented in Figure 4e. O*x*CNT‐based cells exhibited rapid and severe capacity fading (1.82% per cycle), which is attributed to increased film‐like Li_2_S deposition associated with the higher initial capacity (≈1248 mA h g^−1^) compared to ether‐based systems. In contrast, CNT/GQD demonstrated excellent cycling stability (0.77% per cycle) even with its high initial capacity (≈1372 mA h g^−1^), outperforming both CNT (1.39% per cycle and ≈1045 mA h g^−1^, respectively) and O*x*CNT. These findings suggest that a spatially localized introduction of catalytic sites, as in CNT/GQD, is more beneficial for promoting 3D Li_2_S growth, even more pronounced in high donor electrolyte systems than in ether‐based ones. To further evaluate the practicality of our system, cycling tests were conducted under harsh conditions (5 mg_sulfur_ cm^−2^ and E/S = 6.5 µL mg_sulfur_^−1^) using the combination of the CNT/GQD catalyst and the DMI‐based high donor electrolyte, which delivered a high initial charge capacity of 962 mA h g^−1^ and maintained 91.9% of the capacity over 50 cycles, demonstrating excellent performance (Figure S15, Supporting Information).

EIS was used to probe the kinetic properties after discharge (Figure 4f–h). Semi‐circular features were observed in the Nyquist plots for both MWCNT and O*x*CNT substrates after discharge, indicating the presence of residual polysulfides. These results suggest that, similarly to ether‐based electrolyte systems, residual polysulfides are attributed to insufficient catalytic sites in MWCNT and to Li_2_S film–induced passivation in O*x*CNT. These two factors induce sluggish conversion reaction kinetics, thereby leaving a substantial amount of unconverted polysulfides. In contrast, the semicircle vanished for CNT/GQD, implying near‐complete conversion of polysulfides. This observation confirms enhanced reaction kinetics and validates the efficacy of the targeted functional group distribution strategy.

**Figure 5:**
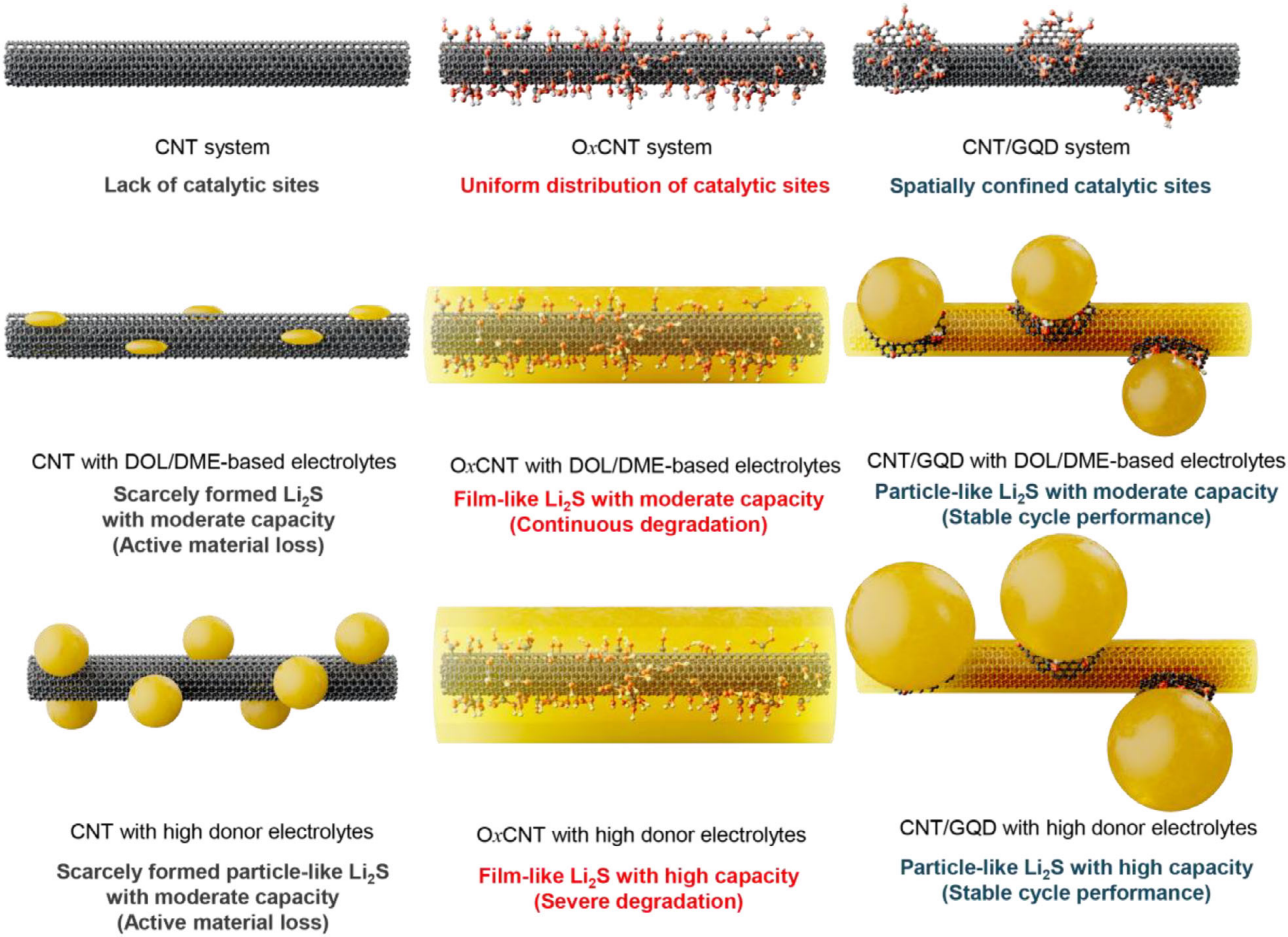
Schematic illustration of morphologies of Li_2_S deposits on CNT, OxCNT, and CNT/GQD substrates with DOL/DME‐based commercial electrolytes and DMI‐based high donor electrolytes. The yellow film‐ or particle‐like materials represent Li_2_S, while the red, white, and black spheres indicate oxygen, hydrogen, and carbon atoms, respectively.

**Figure** **5** illustrates the Li_2_S nucleation behaviors regulated by the spatial distribution of catalytic sites on carbon substrates under different electrolyte systems. The findings obtained in high donor electrolytes offer novel insights that diverge from prior studies, which primarily focused on catalyst design in conventional ether‐based electrolytes or on electrolyte engineering alone. Notably, while the spatial distribution of catalytic sites has previously led to relatively modest impacts on degradation in ether‐based systems, its combination with high donor electrolytes reveals more significant influences on electrochemical performances. This is attributed to the high initial capacities and a larger amount of Li_2_S formation observed in high donor electrolyte systems, where the distinct Li_2_S nucleation mechanisms directly affect surface resistance and, consequently, capacity retention. In such electrolytes, catalysts with uniformly distributed functional groups, such as functionalized carbons or single‐atom catalysts with sub‐nanometer‐scale dispersion, may underperform due to their tendency to promote film‐like Li_2_S growth. In contrast, catalysts with spatially localized active sites, such as particle‐shaped structures, appear more effective in facilitating favorable 3D Li_2_S nucleation, thereby enhancing reaction kinetics and improving cycling stability in advanced electrolyte systems with high sulfur utilization. These observations underscore the importance of tailoring catalyst morphologies and active site distributions to align with the specific solvation environments of the electrolytes.

## 3 Conclusion

In summary, we developed engineered carbon model systems (CNT/GQD and O*x*CNT) to systematically control the spatial distribution of catalytic sites, which profoundly influences Li_2_S nucleation behaviors and polysulfide conversion efficiency in conventional ether‐based electrolytes, and even more pronounced in high donor electrolytes. GQDs, featuring a layered graphene lattice, were incorporated into MWCNTs via π–π interactions. The oxygen‐rich surface of GQDs enhanced sulfiphilicity, enabling strong polysulfide adsorption and catalytic conversion. Furthermore, the integration of GQDs enabled the localized positioning of catalytic sites, which effectively promoted 3D Li_2_S growth and near‐complete polysulfide utilization, under high donor electrolyte conditions as well. These findings deepen the mechanistic understanding of how the spatial distribution of catalytic functionality governs electrochemical performances and degradation. Importantly, the results demonstrate that catalysts with concentrated functional group domains can outperform those with uniform distribution, also within high donor electrolyte systems. This study proposes a broadly applicable strategy for catalyst design in Li–S batteries and provides a framework for improving the efficiency and durability of next‐generation energy storage systems.

## Conflict of Interest

The authors declare no conflict of interest.

## Supporting information



Supporting Information

## Data Availability

The data that support the findings of this study are available from the corresponding author upon reasonable request.
