# Supplementary material for: Exploring the Effects of the Spatial Distribution of Catalytic Sites on Sulfur Nucleation Behaviors and Electrochemical Performances of Lithium–Sulfur Batteries
Source: Adv Sci (Weinh). 2025 Sep 26;12(47):e13026. doi: 10.1002/advs.202513026 (PMC12713044; doi:10.1002/advs.202513026)
Supplement: Supplementary file 1 — Supporting Information [file ADVS-12-e13026-s001.pdf]

Supporting Information

Exploring the Effects of the Spatial Distribution of Catalytic Sites on Sulfur Nucleation Behaviors and Electrochemical Performances of Lithium–Sulfur Batteries

Shin-Yeong Kim[1,2], *Hyeonwoo Cho*[3,4], *Seong-Jun Kim*[1,5], *Minchul Ahn*[3,4], *Yunseo Jeoun*[1,2], *Kookhan Kim*[6], *Sung-Pyo Cho*[7], *So Hee Kim*[8], *Gi Young Son*[3,4], *Seung-Ho Yu*[5,9,*], *Byung Hee Hong*[3,4,*], *Yung-Eun Sung* [1,2,*]

[1] Department of Chemical and Biological Engineering, Seoul National University, Seoul 08826, Republic of Korea

[2] Center for Nanoparticle Research, Institute for Basic Science (IBS), Seoul 08826, Republic of Korea

[3] Department of Chemistry, Seoul National University, Seoul 08826, Republic of Korea

[4] Graphene Research Center, Advanced Institute of Convergence Technology, Suwon 16229, Republic of Korea

[5] Department of Chemical and Biological Engineering, Korea University, Seoul 02841, Republic of Korea

[6] Chemical Materials R&D Department, Korea Automotive Technology Institute, Cheonan, Chungnam 31214, Republic of Korea

[7] National Center for Inter-University Research Facilities, Seoul National University, Seoul 08826, Republic of Korea

[8] Advanced Analysis Center, Korea Institute of Science and Technology (KIST), Seoul 02792, Republic of Korea

[9] Department of Battery-Smart Factory, Korea University, Seoul 02841, Republic of Korea

Shin-Yeong Kim, Hyeonwoo Cho and Seong-Jun Kim contributed equally to this work.

[*]Corresponding author.

A. Experimental section

B. Supplementary Figures (Figure S1–16)

C. Supplementary Tables (Table S1–4)

D. Supplementary Notes (Note S1–5)

A. Experimental section

Synthetic Chemicals: The materials used in this study were as follows: Carbon black (TIMCAL Super C45) was obtained from MTI-Korea. Nitric acid (60% HNO_3) was purchased from DAEJUNG. Anodisc membranes with pore sizes of 20 nm and a diameter of 47 mm, were acquired from Whatman. Multi-walled carbon nanotubes (MWCNTs) were supplied by Carbon Nano-material Technology Co., Ltd. For dialysis, Spectra/Por 7 Dialysis Membrane (molecular weight cutoff of 3.5 kDa) was used.

Preparation of GQDs: Carbon black (900 mg) and 60% nitric acid (240 mL) were added into a two-neck round bottom flask, and the mixture was reacted at 120 °C for 24 hours under sealed conditions. After 24 hours, the sample was further heated at 120 °C for 4 hours to evaporate the nitric acid. Following the evaporation of nitric acid, 20 mL of deionized water was added to the round bottom flask to collect the carbon materials. The collected solution was filtered through 20 nm Anodisc filters. The filtered GQD solution was dialyzed using a 3.5 kDa molecular weight cutoff dialysis membrane for 5 days. The dialyzed solution was freeze-dried to obtain the GQDs.

Preparation of CNT/GQD: 0.8 g of MWCNT and 0.08 g of GQD were added to 180 mL of deionized water and sonicated for 1 hour. The resulting mixture was then freeze-dried to obtain the CNT/GQD composite.

Preparation of O*x*CNT: 0.3 g of MWCNT and 250 mL of 18% w/w HNO_3 were added into round bottom flask and reacted at 140 °C for 24 hours under sealed conditions. After the reaction, the mixture was allowed to stand to facilitate the settling of oxidized MWCNTs. The nitric acid was removed, and the remaining materials were dispersed in deionized water. The O*x*CNT solution was centrifuged at 8000 rpm for 30 minutes. The centrifugation and washing steps were repeated seven times to ensure the removal of residual nitric acid. The purified O*x*CNT was freeze-dried to obtain the final product.

Characterization Techniques: Material characterization was conducted using the following instruments: FT-IR spectra were obtained using a Vertex-80V/Hyperion2000 vacuum FT-IR spectrometer (BRUKER). High-resolution XPS analysis was performed using a Versaprobe 3 system (UL-PHI). Spherical aberration–corrected transmission electron microscopy (Cs-TEM) and bright-field transmission electron microscopy (BF-TEM) imaging were performed on a cold-FEG JEM-ARM200F microscope (Image Cs-corrector, JEOL) at the National Center for Inter-university Research Facilities (NCIRF), Seoul National University (operated at 80 kV for Figures 1d,e,2b, and S3d; at 200 kV for Figure 2c and S3a–c,S6). All images were obtained with a fast and sensitive 16 Megapixel CMOS camera (OneView camera, GATAN), enabling to study atomic scale details by very low-dose and high contrast imaging for beam sensitive samples and low atomic number elements. Dynamic light scattering (DLS) measurements were performed using a Zetasizer Nano ZS instrument (Malvern). Fluorescence measurements were performed using an FP-8300 spectrofluorometer (Jasco Inc.) with excitation at 360 nm, and UV irradiation at 360 nm was applied using a VILBER LOURMAT device. Elemental analysis was conducted using vario MACRO cube (CHN-S), vario MICRO cube (CHN-S), and rapid OXY cube / Flash 2000 (O) analyzers. Raman spectra were obtained using an inVia reflex spectrometer (Renishaw). *Ex-situ* scanning electron microscopy (SEM) was conducted with Regulus 8230 (Hitachi), and samples were transferred through an airtight holder. 4-point probe measurements were conducted using CMT-SR2000N (Advanced Instrument Technology Corp.). Each sample was measured 10 times, and the obtained electrical conductivity values were averaged. *Ex-situ* X-ray diffraction (XRD) patterns were obtained using SmartLab XRD (Rigaku), and samples were transferred through an airtight holder. Prior to all the *ex-situ* analyses, the electrodes were washed three times with 1,3-dioxolane (DOL) solvent (anhydrous, 99.8%, Sigma Aldrich) and subsequently dried overnight in a vacuum chamber.

Li_2S_6 Adsorption Tests: 10 mM Li_2S_6 solutions were prepared by stoichiometrically reacting Li_2S (Sigma Aldrich) and S_8 (Alfa Aesar) in a 1:1 volume ratio mixture of DOL and 1,2-

dimethoxyethane (DME) solvent (anhydrous, 99.5% Sigma Aldrich) at 60 °C for 12 hours. Adsorption tests were conducted by immersing 5 mg of carbon materials in 2 mL of Li_2S_6 solutions for 24 hours.

Electrochemical Measurements: The electrochemical performances were evaluated by fabricating CR2032-type coin cells. The carbon-sulfur composites were prepared by blending carbon substrates (MWCNT, O*x*CNT, CNT/GQD) and elemental sulfur in a weight ratio of 40:60 and heating the blend in an autoclave at 155 °C for 12 hours. Cathodes were prepared by dispersing the carbon/sulfur composite and poly(ethylene oxide) (PEO, Sigma Aldrich, M_w = 600,000) binder in chloroform solvent at a mass ratio of 90:10. Because commonly-used N-methyl-2-pyrrolidinone (NMP) solvent separates GQDs and MWCNTs due to its relatively high polarity, chloroform—a solvent with lower polarity[S1,S2] (Table S4)—was instead selected to maintain their association and to uniformly disperse all the carbon materials used in this study (MWCNT, O*x*CNT, and CNT/GQD) (Figure S16 and Note S4). The as-prepared slurries were cast onto carbon paper (AvCarb P50) using doctor-blade technique. The electrodes were then dried in a convection oven at 60 °C for 12 h. The areal sulfur loading was fixed at ~2 mg_{sulfur} cm^{-2}. Coin cells were fabricated in an Ar-filled glove box by pairing with Li metal discs (Hohsen) as both counter and reference electrodes. 1 M lithium bis(trifluoromethanesulfonyl)imide (LiTFSI) (99.95%, Sigma Aldrich), 0.2 M $LiNO_3$ (99.99%, Sigma Aldrich) in a 1:1 volume ratio mixture of DOL and DME electrolytes and 1 M LiTFSI, 0.2 M $LiNO_3$ in 1,3-dimethyl-2-imidazolidinone (DMI) electrolytes were used, and the relative electrolyte-to-sulfur (E/S) amount was fixed at 20 $\mu L_{electrolyte}$ mg_{sulfur}^{-1}. Galvanostatic charge/discharge measurements and cyclic voltammetry measurements were performed using a potentiostat (WBCS3000 cycler, WonA Tech, Korea), which is installed in a chamber that maintains a constant temperature of 25 °C. Cell voltage range was 1.8-2.8 V vs. Li/Li^+ when DOL/DME-based electrolytes were used, and was 1.7-2.7 V vs. Li/Li^+ when DMI-based electrolytes were used to prevent side reactions. For the preparation of Li_2S-deposited electrodes for *ex-situ* SEM analysis, cathodes with carbon/sulfur composites were discharged

at 0.1C to 1.8 V in DOL/DME-based electrolytes and to 1.7 V in DMI-based electrolytes. Subsequently, the cells were disassembled, and the electrodes were transferred to an airtight holder for SEM analysis. For symmetric CV tests, to match the E/S ratio of 20 μL mg_{sulfur}^{-1}, identical to that used in coin cell tests, the polysulfide concentration was set to 0.25 M. 0.25 M Li_2S_6 catholytes were prepared by stoichiometrically reacting Li_2S and S_8 in DOL/DME-based or DMI-based electrolytes at 60 °C for 12 hours. The electrolyte amount was adjusted so that the sulfur loading corresponded to 1 mg_{sulfur} cm^{-2} for each carbon-only electrode. Voltage scans were carried out between -0.8 V and 0.8 V. For chronoamperometry tests, the sulfur loading corresponded to 1 mg_{sulfur} cm^{-2} for each carbon-only electrode, and E/S ratio was fixed at 20 μL mg_{sulfur}^{-1} using 0.25 M Li_2S_6 catholytes. For the cycle stability test, five initial activation cycles were conducted at 0.1C to redistribute sulfur. Electrochemical impedance spectroscopy (EIS) was measured in a frequency range of 10^{-2}-10^{6} Hz with an amplitude of 10 mV using a potentiostat (ZIVE SP1, WonATech, Korea) at 25 °C.

Statistical Analysis: TEM images (Figures 1d,e,2b,c and S3,S6) were analyzed in DigitalMicrograph (Gatan). Particle sizes were measured in DigitalMicrograph, and distributions were compiled in OriginPro (OriginLab) (Figure S2a). FT-IR (Figures 1b,2d), XPS (Figures 1c,2e, and S5), and Raman (Figure S2b) spectra were acquired in triplicate (n = 3) and processed in OriginPro. Dynamic light scattering (DLS) (Figure S2c) and photoluminescence (PL) spectra (Figures S4b–d) were likewise plotted in OriginPro. XPS peak fitting analysis was performed using the CasaXPS software. Electrochemical measurements data were plotted using OriginPro software.

B. Supplementary Figures

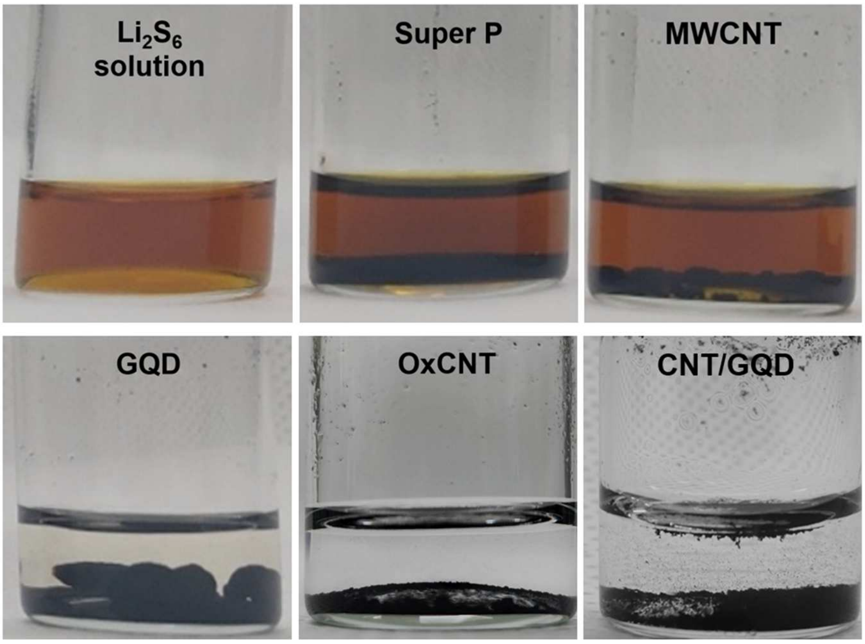

Figure S1. Li_2S_6 adsorption test results of different carbon materials.

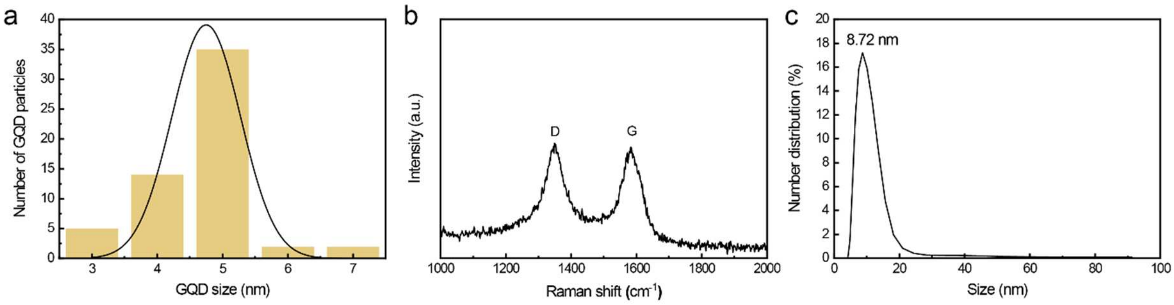

Figure S2. Characterization of GQDs. a) Enlarged size distribution histogram, presented in the inset of Figure 1e. b) Raman spectrum of GQDs. c) DLS analysis of the hydrodynamic diameter of GQDs.

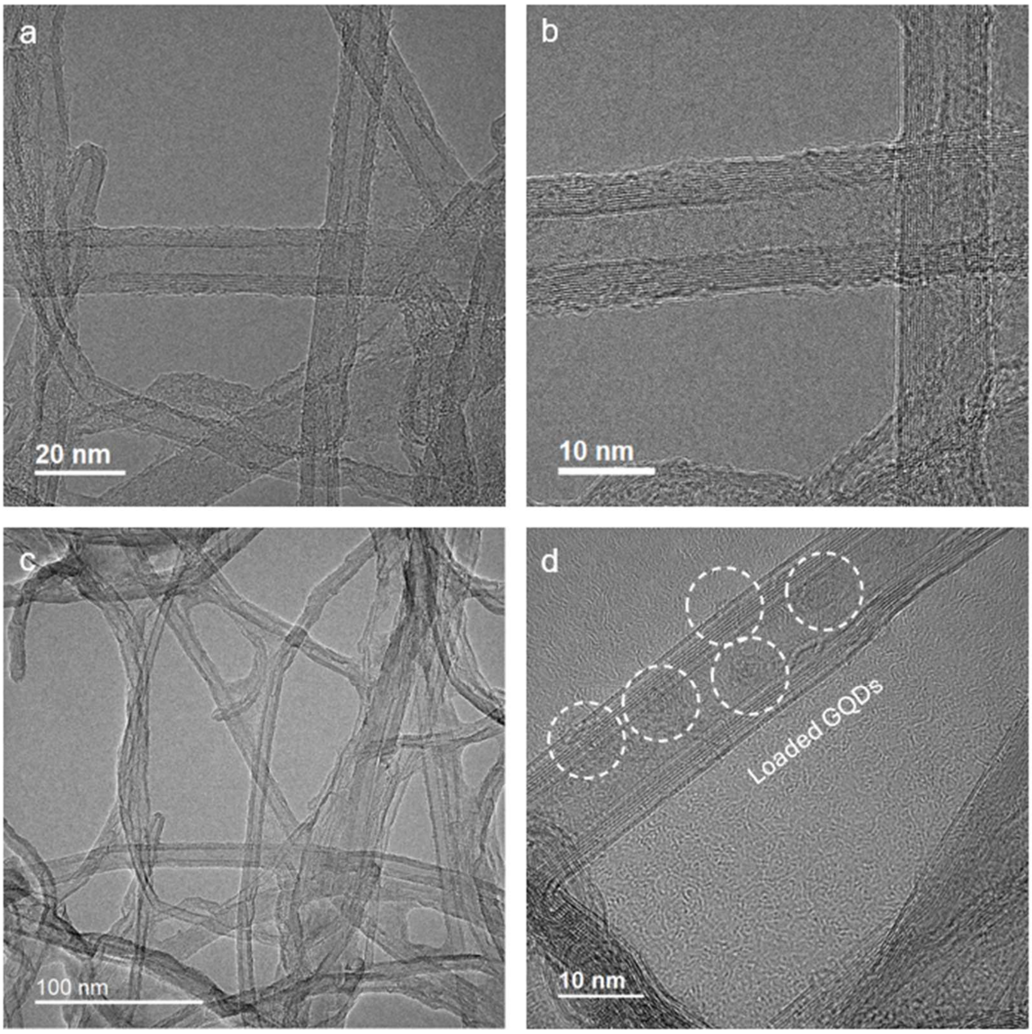

Figure S3. Morphological characterization of carbon materials. a–c) Representative Cs-TEM images of MWCNT at three distinct regions. d) The Cs-TEM image of CNT/GQDs.

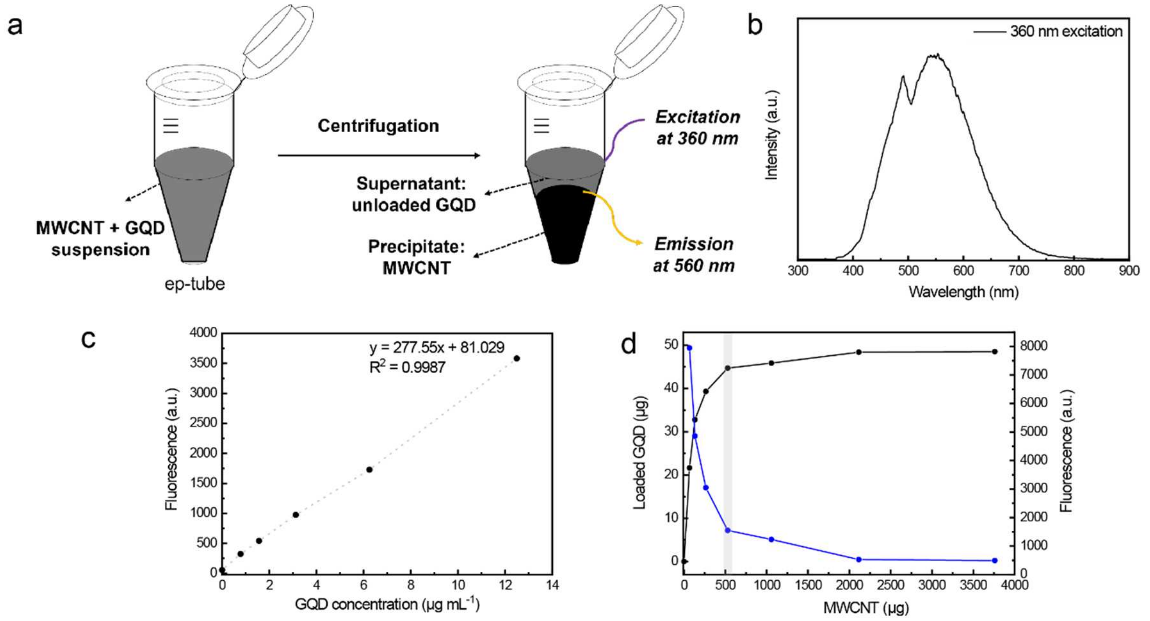

Figure S4. Characterization of maximum loading amount of GQDs on MWCNTs. a) Schematic diagram of photoluminescence (PL) measurement. b) PL spectrum of GQDs excited at 360 nm. Inset photograph displays the fluorescence of GQDs under 360 nm excitation. c) Standard curve established by correlating fluorescence intensities with GQD concentrations. d) Quantification of GQD loading amounts during the process of preparing CNT/GQDs. 50 μg of GQDs were introduced to dispersion solutions with different amounts of MWCNTs. When more than 500 μg of MWCNTs were present in dispersion solutions, nearly all the 50 μg of GQDs were loaded. Black; Loaded GQDs, Blue; Fluorescence.

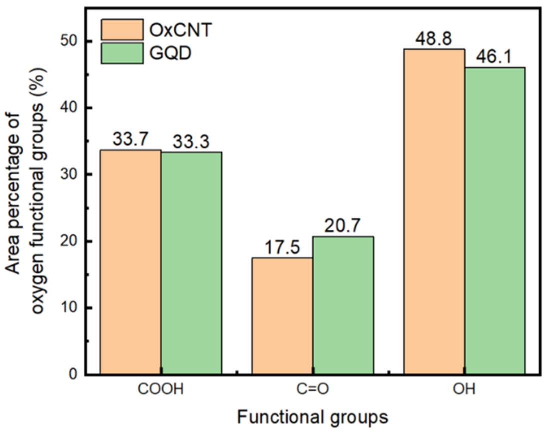

Figure S5. Comparison of the area percentages of oxygen functional groups using C 1s XPS results.

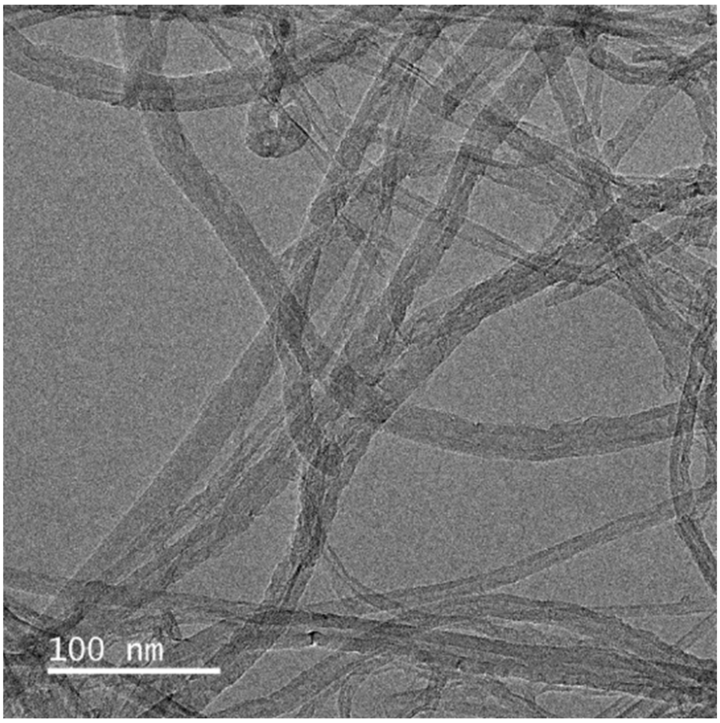

Figure S6. Transmission electron microscopy (TEM) image of O*x*CNTs.

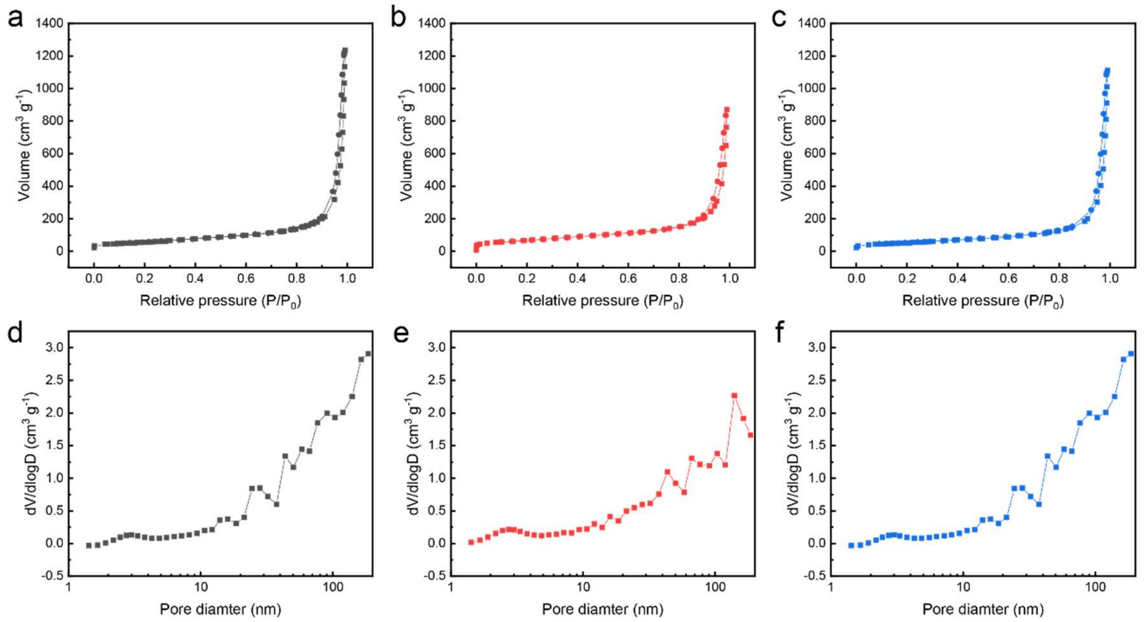

Figure S7. N_2 adsorption/desorption isotherms of a) MWCNT, b) O*x*CNT, and c) CNT/GQD. Pore size distributions of d) MWCNT, e) O*x*CNT, and f) CNT/GQD.

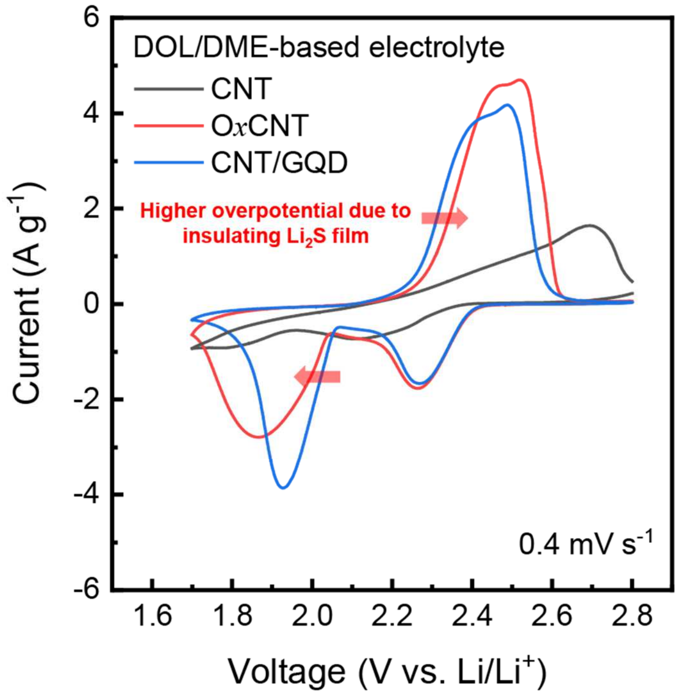

Figure S8. Cyclic voltammetry results of carbon substrates in DOL/DME-based electrolytes.

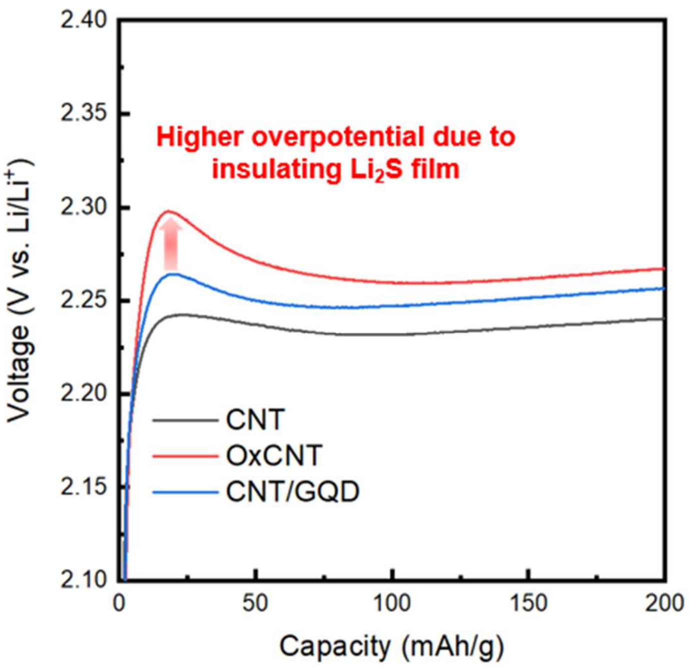

Figure S9. Amplified voltage profiles of the initial galvanostatic charging processes.

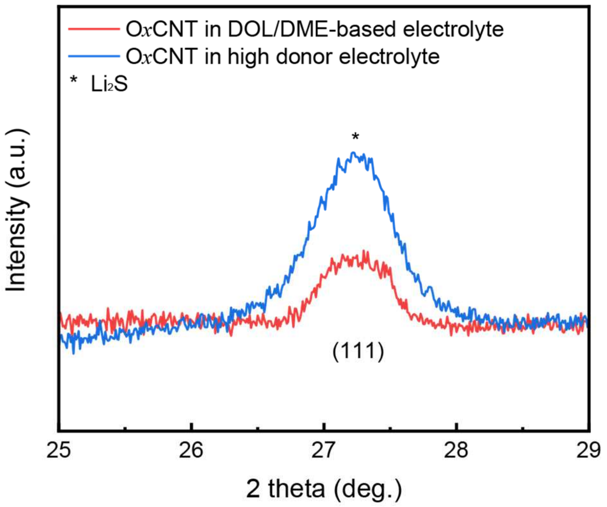

Figure S10. *Ex-situ* XRD patterns of discharged state of O*x*CNT cathodes in DOL/DME-based electrolyte and high donor electrolyte.

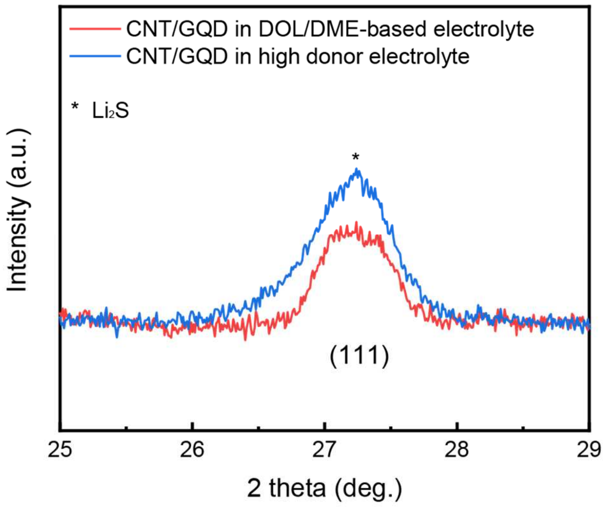

Figure S11. *Ex-situ* XRD patterns of discharged state of CNT/GQD cathodes in DOL/DME-based electrolyte and high donor electrolyte.

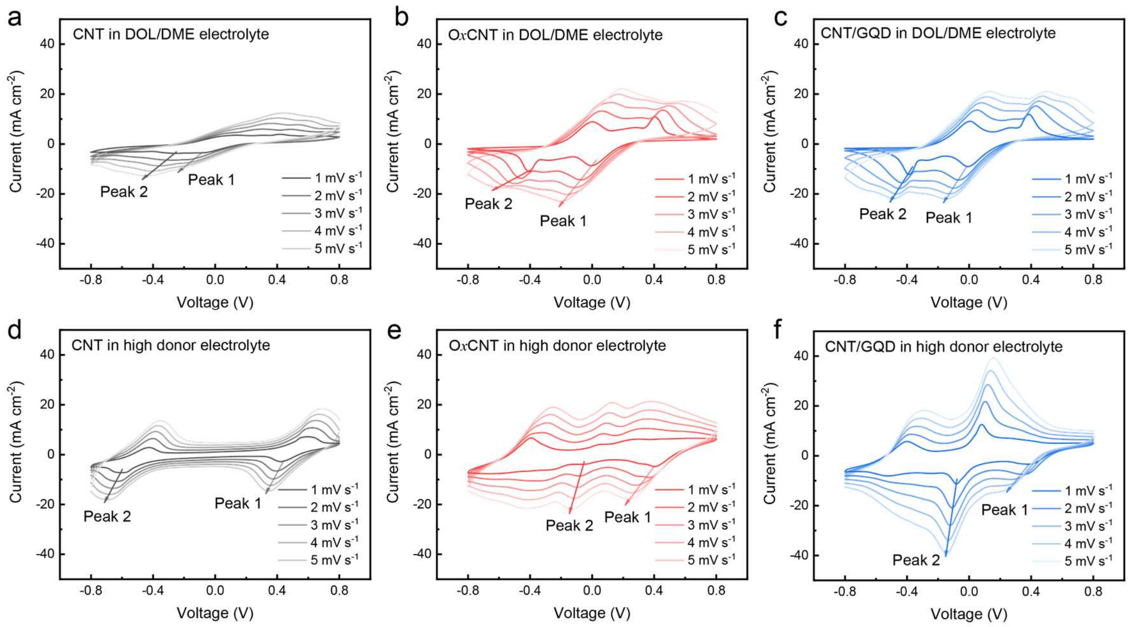

Figure S12. Symmetric CV tests of a) CNT, b) O*x*CNT, and c) CNT/GQD in DOL/DME-based electrolyte., and d) CNT, e) O*x*CNT, and f) CNT/GQD in DMI-based electrolyte.

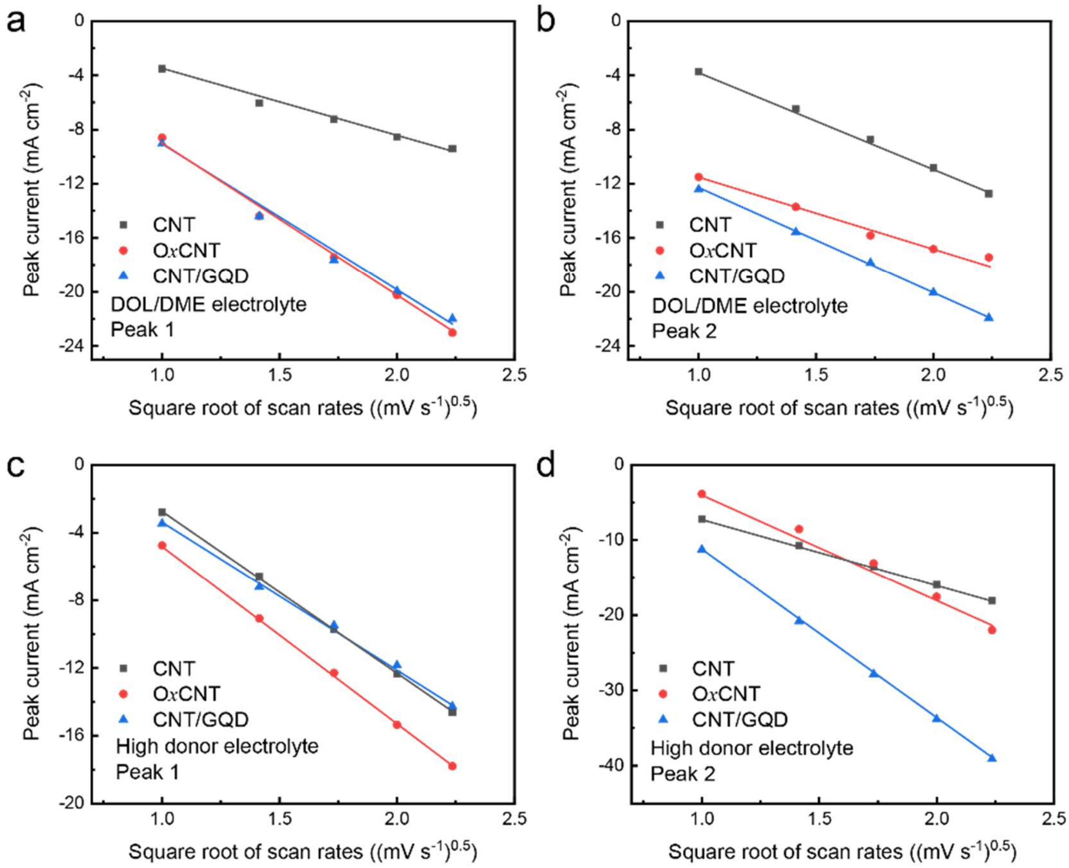

Figure S13. Plots of peak current (i_P) versus the square root of scan rate ($\nu^{0.5}$) obtained from symmetric CV tests: (a) Peak 1 and (b) Peak 2 in the DOL/DME-based cell, and (c) Peak 1 and (d) Peak 2 in the DMI-based cell.

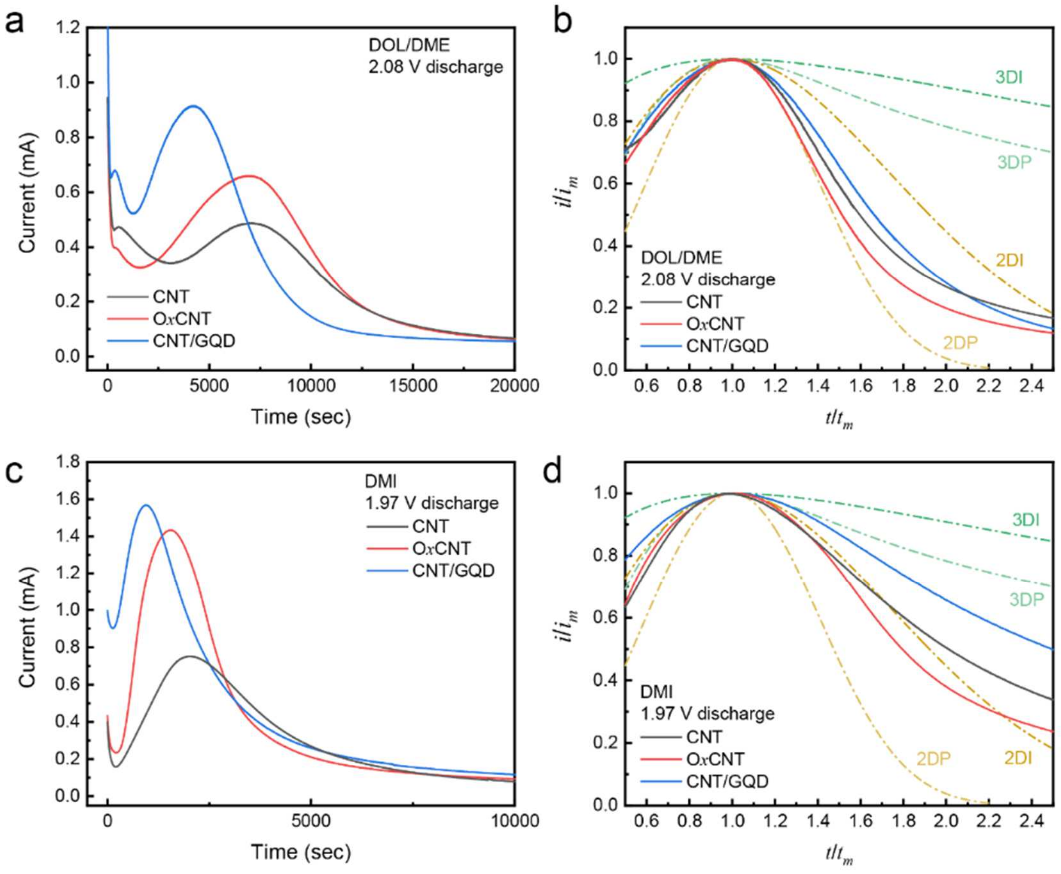

Figure S14. Chronoamperometry test results for a) DOL/DME-based electrolytes and b) the corresponding fitted dimensionless values for elucidating nucleation mechanisms; and for c) DMI-based electrolytes and d) the corresponding fitted dimensionless values for elucidating nucleation mechanisms.

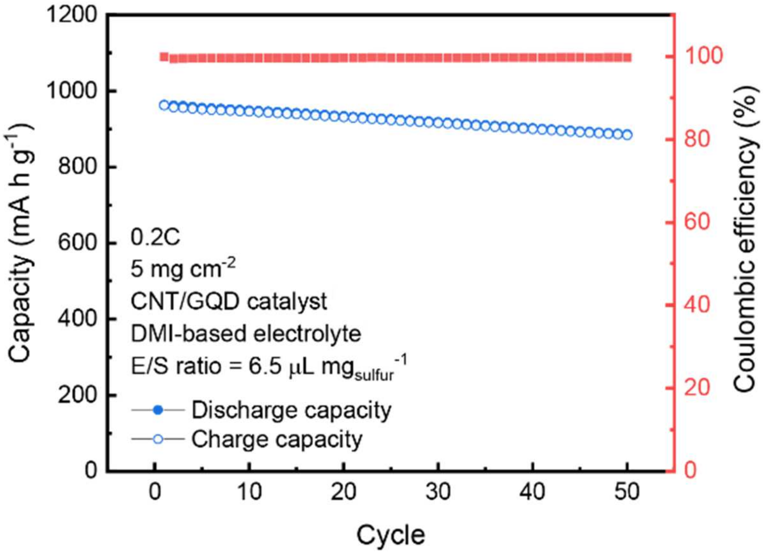

Figure S15. Cycling test results under harsh conditions using the CNT/GQD catalyst with the DMI-based high donor electrolyte.

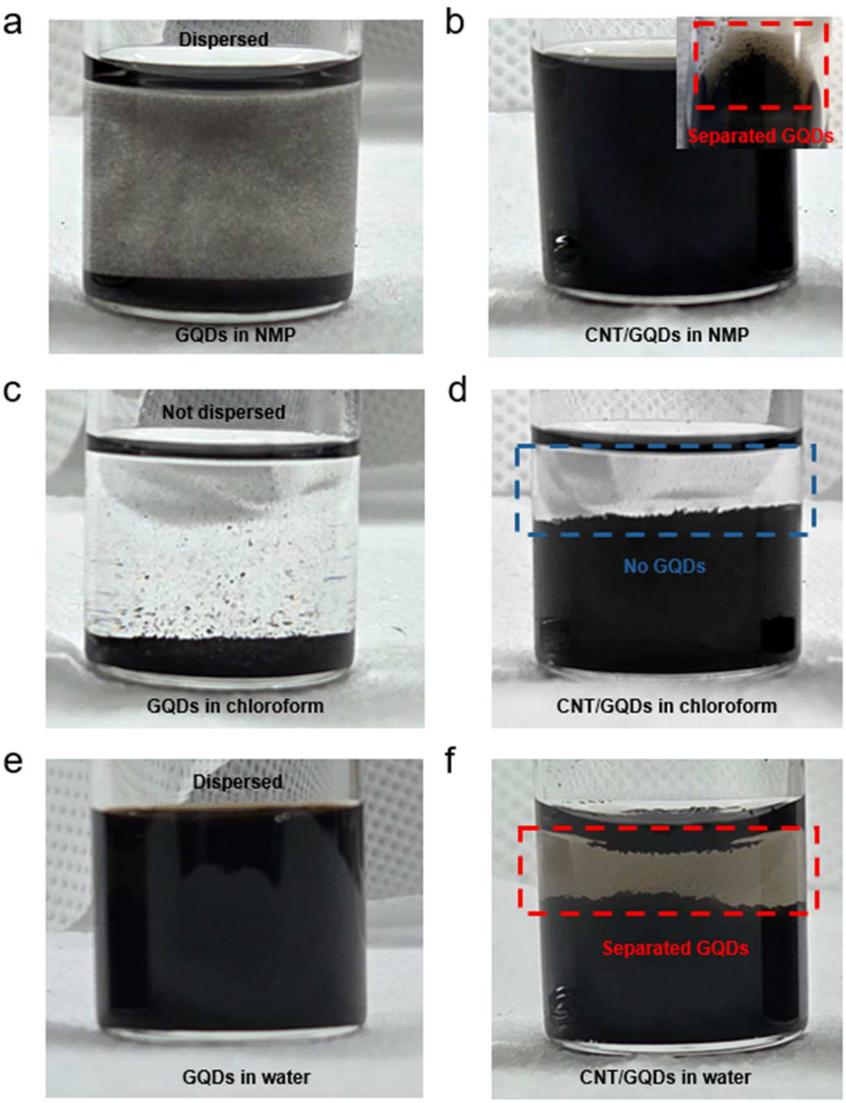

Figure S16. Photographs of a) GQD dispersion solution and b) CNT/GQD dispersion solution in NMP solvent. The inset of (b) shows the darkened color arising from separated GQDs in the NMP supernatant. Photographs of c) GQD dispersion solution and d) CNT/GQD dispersion solution in chloroform solvent. Photographs of e) GQD dispersion solution and f) CNT/GQD dispersion solution in water solvent. For each CNT/GQD dispersion, 50 mg of CNT/GQD powder was immersed in 5 mL of solvent, while for each GQD dispersion, 5 mg of freeze-dried GQDs (corresponding to a CNT:GQD weight ratio of 10:1) was immersed in 5 mL of solvent.

C. Supplementary Tables

Elemental analysis results

(Unit: wt%)

Sample name	Carbon	Hydrogen	Oxygen	Nitrogen
GQD	47.561	2.297	49.511	0.631
CNT	98.300	0.422	0.978	0.300
CNT/GQD (10:1 w/w)*	93.226	0.610	5.831	0.333
O*x*CNT	90.281	0.878	6.610	0.319

* Theoretical oxygen content calculated from the weight ratio between CNT and GQD

Table S1. Elemental analysis results of carbon materials.

Materials	XPS C 1s peak fitted (Unit: eV)						Reference
	C=C (sp^2)	C-C (sp^3)	C-OH	C-O-C	C=O	COOH	
Graphene oxide (GO)	284.6	-	285.6	286.7	288.2	289.4	[S3]
Graphene, GO, reduced graphene oxide (rGO)	284.4	285.0	285.7	286.7	288.0	289.1	[S4]
Graphene	284.6	-	285.5	286.4	287.6	288.8	[S5]
GO	284.47	284.85	286.55	287.05	288.05	289.05	[S6]
GO	284.5	-	285.86	286.55	287.54	288.94	[S7]
GO	284.6	284.9	285.9	286.9	288.2	289.3	[S8]
Graphene	284.6	-	285.7	286.6	287.5	288.7	[S9]
rGO	284.8	-	285.9	286.6	287.5	288.9	[S10]
GO	284.5	285.4	286.5	287.2	288.5	-	[S11]
GO	284.6	-	286.4	287.1	288.7	-	[S12]
rGO			286.2	287.0	288.6		
GO	284.1	285.0	285.7	286.7	288.0	289.0	[S13]
rGO	284.6	-	285.6	286.7	288.2	289.4	[S14]
GO, rGO	284.5	285.3	286.3	287.1	288.2	289.4	[S15]
GO	284.6	-	286.7	287.0	288.2	288.9	[S16]
CNT	284.8	-	286.3	286.9	288.8	290.1	[S17]
CNT	284.8		285.9	286.9	288.2	289.3	[S18]
CNT	284.8		285.9	286.9	288.2	289.3	[S19]
CNT	284.8		285.9	286.9	288.2	289.3	[S20]
CNT	284.5		285.6	286.5	287.8	288.9	[S21]
CNT	284.5	284.9	285.7	286.7	288.8	289.3	[S22]

Table S2. Literature-reported C 1s peak positions (eV) for oxygen-containing functional groups in oxidized graphene–like sp^2 carbons. Values are extracted as reported.

4-point probe results

(Unit: S/m)

Electrode	Electrical conductivity
CNT	6.725×10^4
O*x*CNT	6.610×10^4
CNT/GQD	6.691×10^4

Table S3. 4-point probe results for electrodes with different carbon substrates.

Snyder’s polarity index values for various solvents

Solvent	Snyder’s polarity index
Pentane	0.0
Dichloromethane	3.1
2-Propanol (IPA)	3.9
**Chloroform**	**4.1**
Methanol	5.1
Acetonitrile	5.8
N,N-Dimethylformamide (DMF)	6.4
**NMP**	**6.7**
Dimethyl Sulfoxide (DMSO)	7.2
Water	10.2

Table S4. Comparison of Snyder’s polarity index values for various solvents.[S1]

D. Supplementary Notes

Note S1. Rationales for XPS devolution processes.

Numerous studies have reported that, in the C 1s region, the binding energy (BE) of C–OH is comparable to or higher than that of C–O–C.[S33] At the same time, in oxidized graphene–like (sp^2) substrates, there are reports in which the BE of C–O–C (epoxy) lies slightly higher than that of C–OH (hydroxyl) under specific structural/chemical contexts. For example, in graphene oxide (GO), the oxidized envelope (~286–289 eV) was deconvoluted into epoxy at ~287.05 eV and hydroxyl at ~286.55 eV. Density functional theory (DFT) chemical-shift calculations reproduced this order (mean ΔBE: epoxy (+2.36 eV) > hydroxyl (+2.29 eV)). Moreover, cyclic voltammetry results showed hydroxyl reduction at a more negative potential than epoxy (~0.3 V), supporting the assignment of epoxy at the higher BE.[S6] In a related sp^2 substrate (CNT-modified ultrathin CF/PET tape), high-resolution C 1s spectra likewise assigned C–OH at 286.3 eV and C–O–C at 286.9 eV, reaffirming the higher BE of epoxy.[S17]

Against this background, our GQD and O*x*CNT samples are sp^2-based nanocarbons bearing oxygen functionalities and therefore share an electronic/structural context with oxidized graphene. Applying an identical fitting protocol to both samples (GL(14) for C=C (sp^2), GL(0) for other components; FWHM 0.6–1.3 eV; charge-referenced to sp^2 C 1s at 284.8 eV with a Shirley background), we obtained the following results: GQD, C–OH at 286.1 eV, C–O–C at 287.1 eV (ΔBE = +1.0 eV); O*x*CNT, C–OH at 286.3 eV, C–O–C at 287.0 eV (ΔBE = +0.7 eV). These separations fall within the oxidized envelope and are consistent with the C 1s component assignments reported in the cited literature.

Physically, basal-plane epoxides (C–O–C, three-membered rings) place each ring carbon in a sp^3-like (out-of-plane) environment with ring strain and insert two C–O bonds within the same local π-domain, thereby imposing stronger π-network disruption. The resulting lower initial-state electron density and weaker final-state screening lead to a slightly higher C 1s BE than for basal hydroxyls (C–OH), which typically preserve better π-coupling to the surrounding sp^2 lattice. This mechanism is consistent with the XPS/DFT/CV cross-validation in the cited study.[S6]

Note S2. EIS fitting.

We have attempted to use equivalent circuit fitting from the early stages of this study. However, in the case of Li–S batteries, the cell conditions between the pristine and discharge-end states are markedly different, particularly in terms of morphology. In our experiments, this discrepancy made it difficult to establish a single equivalent circuit model applicable across all samples and states of charge/discharge; instead, each condition would require a distinct equivalent circuit model. Although we referred to previous reports, we found insufficient basis to define one universal model. In fact, even when we followed the fitting approaches reported in the literature, the results were not satisfactory, which indicated the need for constructing novel and more complex models. However, we found that several recent reports adopting EIS analysis approaches that only track the variation of a single semicircle (corresponding to R_{CT}), rather than relying on complete equivalent circuit fitting.[3,42] Accordingly, we followed this approach in this work.

Note S3. Symmetric CV tests.

Additional kinetic analyses were performed using symmetric CV tests.[S23] During the symmetric CV measurements, we particularly focused on resolving the responses into two redox peaks, designated as Peak 1 and Peak 2. In a symmetric configuration, when the working electrode undergoes the sulfur reduction reaction, the counter electrode simultaneously undergoes the sulfur oxidation reaction. However, sulfur oxidation in a conventional Li–S cell is typically complex and occurs over a broad potential range without clear peak separation. Thus, the peaks observed in the symmetric CV can be reasonably attributed to the distinct steps of the sulfur reduction process, analogous to the cathodic reactions of CV tests in a Li–S full cell. Specifically, Peak 1 corresponds to the reduction of S_8 to Li_2S_8 (solid-to-liquid transition), whereas Peak 2 is associated with the further reduction of Li_2S_4 to Li_2S (liquid-to-solid transition).

While monitoring how Peaks 1 and 2 evolve with scan rate, we observed that in DOL/DME-based electrolyte, the integration of O*x*CNT and CNT/GQD catalysts led to stronger redox peaks with increased current for both Peak 1 and Peak 2 (Figure S12a–c), indicating that the catalysts enhance sulfur conversion kinetics. Notably, the Peak 2 current was significantly higher for CNT/GQD compared to O*x*CNT. Since Peak 2 corresponds to Li_2S nucleation, this suggests that CNT/GQD exhibits superior Li_2S nucleation kinetics, likely facilitated by its 3D morphology, which promotes Li_2S growth and thus improves the overall kinetics.

In contrast, in the high donor DMI electrolyte, Peak 1 did not show notable enhancement, but Peak 2 for CNT/GQD exhibited a much stronger redox response compared to O*x*CNT (Figure S12d–f). This indicates that the improvement in Li_2S nucleation kinetics is more pronounced in the DMI electrolyte, consistent with our finding that localizing catalytic sites is a more effective strategy in high donor electrolytes.

To further evaluate Li^+ ion transport behavior at Peaks 1 and 2 in each electrolyte, the Randles–Sevcik equation was applied to compare diffusion coefficients. The peak current (i_p)

exhibited a linear correlation with the square root of the scan rate ($\nu^{0.5}$), as expected from the Randles–Sevcik relationship. The governing equation is expressed as:

$$i_p = 0.4463\, n^{3/2} F^{3/2} A \frac{D^{1/2} c v^{1/2}}{(RT)^{1/2}}$$

In the Randles–Sevcik equation, n is the number of electrons transferred in the redox process, F is the Faraday constant, A is the electrode surface area, D is the diffusion coefficient of the electroactive species, C is its bulk concentration, R is the gas constant, and T is the absolute temperature. According to this relationship, plotting i_p against $v^{0.5}$ yields a linear dependence, where the slope is proportional to the square root of the diffusion coefficient. As shown in Figure S13, the plots were obtained for our system. While the diffusion coefficients derived from Peak 1 exhibited negligible differences, those from Peak 2 were significantly larger for CNT/GQD compared to O*x*CNT, implying once again that the CNT/GQD catalyst enhances Li_2S nucleation kinetics.

Note S4. Chronoamperometry tests.

To investigate the growth kinetics and mechanism of Li_2S, chronoamperometry nucleation experiments were conducted. The chronoamperometry results obtained in the DOL/DME-based electrolyte are shown in Figure S14a. Compared with CNT, both O*x*CNT and CNT/GQD exhibited higher peak currents owing to the introduction of oxygen functional groups. Notably, CNT/GQD displayed a sharper and earlier peak than O*x*CNT, indicating superior Li_2S nucleation kinetics. Furthermore, the current–time profiles from chronoamperometry can be analyzed to distinguish the type of nucleation tendency by comparison with four classical models. These models include the Scharifker–Hills (SH) models—(1) three-dimensional instantaneous (3DI) and (2) three-dimensional progressive (3DP) nucleation, which involve bulk-diffusion-controlled growth—and the Bewick–Fleischman–Thirsk (BFT) models—(3) two-dimensional instantaneous (2DI) and (4) two-dimensional progressive (2DP) nucleation, which involve growth controlled by the incorporation of adatoms into the lattice.[S24-28] The mathematical expressions describing these four models are given below.

SH model 1) 3DI model:

$$\frac{I^2}{{I_m}^2} = \frac{1.9542}{t/t_m}\{1 - \exp\left[-1.2564\left(\frac{t}{t_m}\right)\right]\}^2$$

SH model 2) 3DP model:

$$\frac{I^2}{{I_m}^2} = \frac{1.2254}{t/t_m}\{1 - \exp\left[-2.3367\left(\frac{t}{t_m}\right)^2\right]\}^2$$

BFT model 1) 2DI model:

$$\frac{I}{I_m} = \frac{t}{t_m}\exp\{\frac{1}{2}\left[1 - \left(\frac{t}{t_m}\right)^2\right]\}$$

BFT model 2) 2DP model:

$$\frac{I}{I_m} = (\frac{t}{t_m})^2\ \exp\{\frac{2}{3}\left[1 - \left(\frac{t}{t_m}\right)^3\right]\}$$

Both time and current responses were normalized into dimensionless values and plotted as shown in Figure S14b. Examination of the nucleation tendencies revealed that CNT and OxCNT fit better with the 2DP model. In contrast, CNT/GQD showed a trend approaching 3D growth models; the thin two-dimensional film remaining on the CNT/GQD surface coexisted with the onset of three-dimensional nucleation, indicating that the original 2DP mode was partially retained but shifted toward 3DP with the incorporation of CNT/GQD. These behaviors are recognized in previous reports as indicative of mixed 2D/3D growth.[S29,S30]

In the case of the DMI electrolyte, the chronoamperometry results are presented in Figure S14c. Similar to the DOL/DME-based system, both O*x*CNT and CNT/GQD exhibited higher peak currents than CNT, with CNT/GQD reaching its peak current at an earlier time, confirming faster Li_2S nucleation kinetics. When comparing the current–time profiles with the classical nucleation models (Figure S14d), CNT and O*x*CNT appeared closer to 2DI. However, considering the mixed features of 2DP and 3DP observed in the fitting results, the interpretation that these electrodes exhibited combined 2DP/3DP behavior. Importantly, CNT/GQD displayed features closer to 3DP, suggesting that the introduction of CNT/GQD promoted three-dimensional growth of Li_2S.

Note S5. Rationales for choosing chloroform as a solvent for slurry casting.

At first, CNT/GQD composites were dispersed in the commonly used NMP solvent for slurry casting. However, we observed that the supernatant of NMP dispersion darkened, a phenomenon likewise observed when GQDs were dispersed in NMP alone (Figures S16a,b). This indicates that GQDs detach from CNT surfaces during dispersion, motivating the search for an alternative solvent that better preserves the CNT–GQD composite structure. The detachment primarily arises because the interaction between GQDs and the solvent becomes stronger than the π–π interactions anchoring GQDs onto the MWCNT surface. To suppress detachment of GQDs, the relative strength of GQD–solvent interactions should be carefully considered.

Given that GQDs are rich in oxygen-containing functional groups, they possess strong polarity and thus tend to interact favorably with highly polar solvents, which enhances dispersion of GQDs. Therefore, to reduce GQD detachment into the solvent, solvent with lower polarity than NMP should be employed. Based on Snyder's polarity index values (Table S4), chloroform can be considered as a potential candidate solvent. In this study, we selected chloroform due to its compatibility with PEO, a commonly used polymer binder reported in prior studies.[S31,S32] Although chloroform is toxic, the same strategy of lowering solvent polarity could be extended to other solvents, provided a suitable binder is selected.

As shown in Figure S16c, when using chloroform (lower polarity than NMP), GQD–solvent polar-polar interactions were significantly reduced, and GQDs were not dispersed into the solvent. Likewise, immersing CNT/GQD in chloroform did not result in GQD detachment (Figure S16d). In contrast, when water (a more polar solvent than NMP) was employed, strong polar-polar interactions stabilized the dispersion of GQDs in solution (Figure S16e). Consequently, the supernatant of the CNT/GQD immersion solution became dark, indicating the separation of GQDs into the water solution (Figure S16f).

References

[S1] L. R. Snyder, *Journal of Chromatographic Science* **1978**, 16, 223.
[S2] X. Huang, F. Cao, S. Zhan, Q. Feng, M. Zhu, Z. Su, X. Gao, J. Yin, J. Li, N. Zheng, B. Wu, *Joule* **2023**, 7, 1556.
[S3] S. Park, K.-S. Lee, G. Bozoklu, W. Cai, S. T. Nguyen, R. S. Ruoff, *ACS Nano* **2008**, 2, 572.
[S4] A. Kovtun, D. Jones, S. Dell'Elce, E. Treossi, A. Liscio, V. Palermo, *Carbon* **2019**, 143, 268.
[S5] R. Singla, A. Kottantharayil, *Carbon* **2019**, 152, 267.
[S6] I. Ferrari, A. Motta, R. Zanoni, F. A. Scaramuzzo, F. Amato, E. A. Dalchiele, A. G. Marrani, *Carbon* **2023**, 203, 29.
[S7] A. Ganguly, S. Sharma, P. Papakonstantinou, J. Hamilton, *The Journal of Physical Chemistry C* **2011**, 115, 17009.
[S8] F. Perrozzi, S. Prezioso, M. Donarelli, F. Bisti, P. De Marco, S. Santucci, M. Nardone, E. Treossi, V. Palermo, L. Ottaviano, *The Journal of Physical Chemistry C* **2013**, 117, 620.
[S9] R. Hawaldar, P. Merino, M. R. Correia, I. Bdikin, J. Grácio, J. Méndez, J. A. Martín-Gago, M. K. Singh, *Scientific Reports* **2012**, 2, 682.
[S10] L. Li, Q. Liu, Y.-X. Wang, H.-Q. Zhao, C.-S. He, H.-Y. Yang, L. Gong, Y. Mu, H.-Q. Yu, *Scientific Reports* **2016**, 6, 30082.
[S11] N. Bandara, Y. Esparza, J. Wu, *Scientific Reports* **2017**, 7, 11538.
[S12] W. Pang, J. Xue, H. Pang, *Scientific Reports* **2019**, 9, 5224.
[S13] K. Z. Donato, H. L. Tan, V. S. Marangoni, M. V. S. Martins, P. R. Ng, M. C. F. Costa, P. Jain, S. J. Lee, G. K. W. Koon, R. K. Donato, A. H. Castro Neto, *Scientific Reports* **2023**, 13, 6064.
[S14] I. K. Moon, J. Lee, H. Lee, *Chemical Communications* **2011**, 47, 9681.
[S15] J. Liu, T. Yan, Y. Li, H. Ren, Q. Wang, F. Guan, Q. Jiao, *RSC Advances* **2020**, 10, 10789.
[S16] M. K. Rabchinskii, V. V. Shnitov, M. Brzhezinskaya, M. V. Baidakova, D. Y. Stolyarova, S. A. Ryzhkov, S. D. Saveliev, A. V. Shvidchenko, D. Y. Nefedov, A. O. Antonenko, S. V. Pavlov, V. A. Kislenko, S. A. Kislenko, P. N. Brunkov, *Nanomaterials* **2023**, 13, 23.
[S17] G. Yang, J. Cui, S. Zhang, X. Kuang, Y. Luo, F. Bao, J. Yu, H. Liu, C. Zhu, J. Xu, *Composites Part B: Engineering* **2024**, 284, 111718.
[S18] A. A. D'Archivio, M. A. Maggi, A. Odoardi, S. Santucci, M. Passacantando, *Nanotechnology* **2018**, 29, 065701.
[S19] L. Camilli, D. Capista, P. Eramo, A. A. D'Archivio, M. A. Maggi, A. Lazzarini, M. Crucianelli, M. Passacantando, *Nanotechnology* **2022**, 33, 245707.
[S20] B. M. Bizzarri, I. Abdalghani, L. Botta, A. R. Taddei, S. Nisi, M. Ferrante, M. Passacantando, M. Crucianelli, R. Saladino, *Nanomaterials* **2018**, 8, 516.
[S21] A. N. Redkin, A. A. Mitina, E. E. Yakimov, E. N. Kabachkov, *Materials* **2021**, 14, 7612.
[S22] V. Golovakhin, E. Y. Kim, O. N. Novgorodtseva, E. A. Maksimovskiy, A. V. Ukhina, A. V. Ishchenko, A. G. Bannov, *Membranes* **2023**, 13, 729.
[S23] X. Zuo, M. Zhen, D. Liu, H. Yu, X. Feng, W. Zhou, H. Wang, Y. Zhang, *Advanced Functional Materials* **2023**, 33, 2214206.
[S24] P. Wang, B. Xi, M. Huang, W. Chen, J. Feng, S. Xiong, *Advanced Energy Materials* **2021**, 11, 2002893.
[S25] Z. Guan, X. Chen, F. Chu, R. Deng, S. Wang, J. Liu, F. Wu, *Advanced Energy Materials* **2023**, 13, 2302850.

[S26] B. Scharifker, G. Hills, *Electrochimica Acta* **1983**, 28, 879.
[S27] F. Y. Fan, W. C. Carter, Y.-M. Chiang, *Advanced Materials* **2015**, 27, 5203.
[S28] A. Milchev, I. Krastev, *Electrochimica Acta* **2011**, 56, 2399.
[S29] Y.-X. Yao, J. Wan, N.-Y. Liang, C. Yan, R. Wen, Q. Zhang, *Journal of the American Chemical Society* **2023**, 145, 8001.
[S30] J.-L. Yang, D.-Q. Cai, Q. Lin, X.-Y. Wang, Z.-Q. Fang, L. Huang, Z.-J. Wang, X.-G. Hao, S.-X. Zhao, J. Li, G.-Z. Cao, W. Lv, *Nano Energy* **2022**, 91, 106669.
[S31] W. J. Chung, J. J. Griebel, E. T. Kim, H. Yoon, A. G. Simmonds, H. J. Ji, P. T. Dirlam, R. S. Glass, J. J. Wie, N. A. Nguyen, B. W. Guralnick, J. Park, Á. Somogyi, P. Theato, M. E. Mackay, Y.-E. Sung, K. Char, J. Pyun, *Nature Chemistry* **2013**, 5, 518.
[S32] C. Man, P. Jiang, K.-w. Wong, Y. Zhao, C. Tang, M. Fan, W.-m. Lau, J. Mei, S. Li, H. Liu, D. Hui, *Journal of Materials Chemistry A* **2014**, 2, 11980.
[S33] J. Kerber, J. J. Bruckner, K. Wozniak, S. Seal, S. Hardcastle, T. L. Barr, *Journal of Vacuum Science & Technology A* **1996**, 14, 1314.
